# Supplementary figures and images for: The conserved σD envelope stress response monitors multiple aspects of envelope integrity in corynebacteria
Source: PLoS Genet. 2024 Jun 3;20(6):e1011127. doi: 10.1371/journal.pgen.1011127 (PMC11175481; doi:10.1371/journal.pgen.1011127)

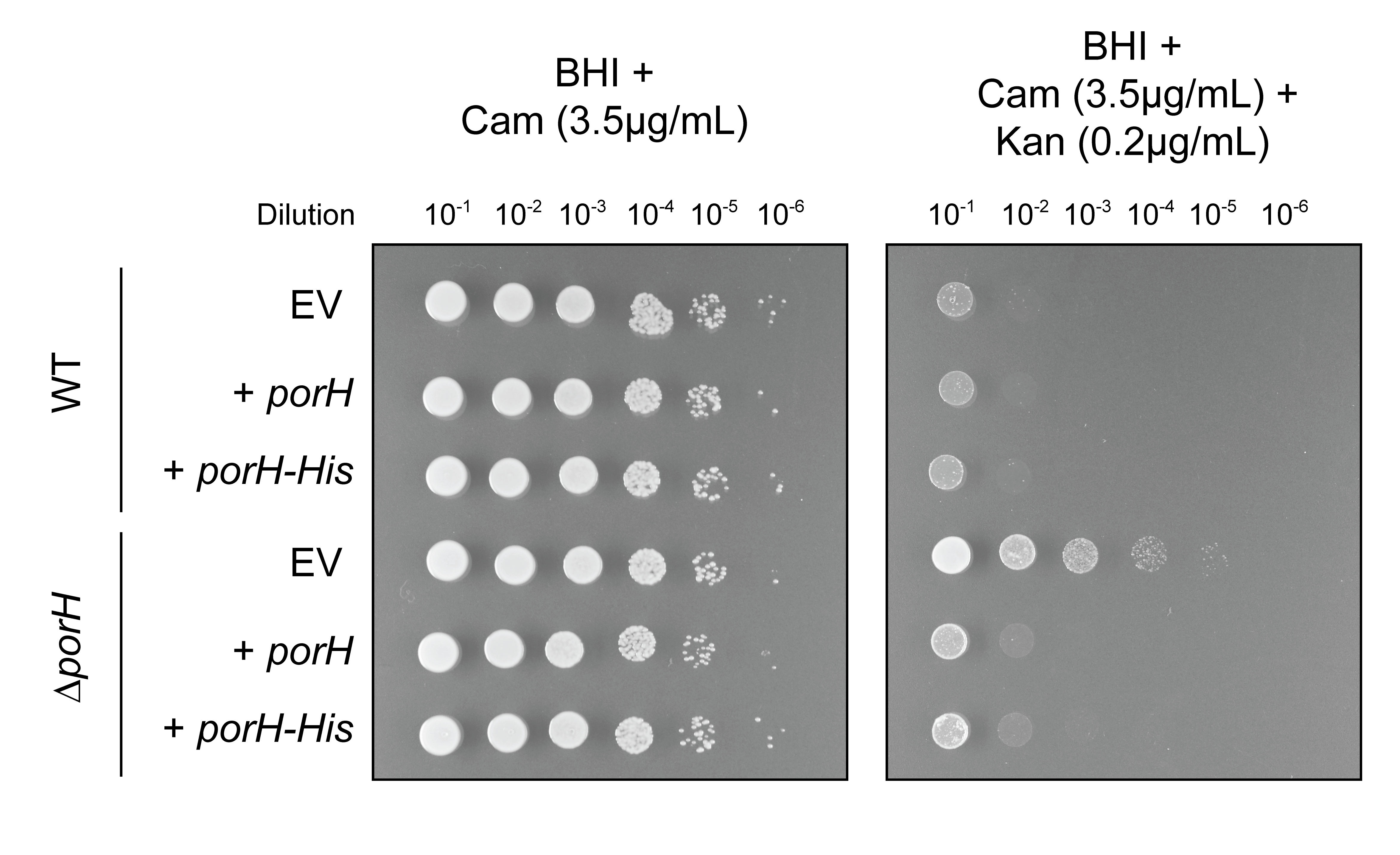

Supplement: S1 Fig — Spot titers testing kanamycin sensitivity were prepared by making ten-fold serial dilutions of the indicated cultures and spotting onto media with or without the addition of kanamycin. WT or ΔporH cells harbored either an empty vector (EV) or plasmids constitutively expressing PorH or PorH-His. Chloramphenicol was included for plasmid maintenance. (TIF) [file pgen.1011127.s001.tif]

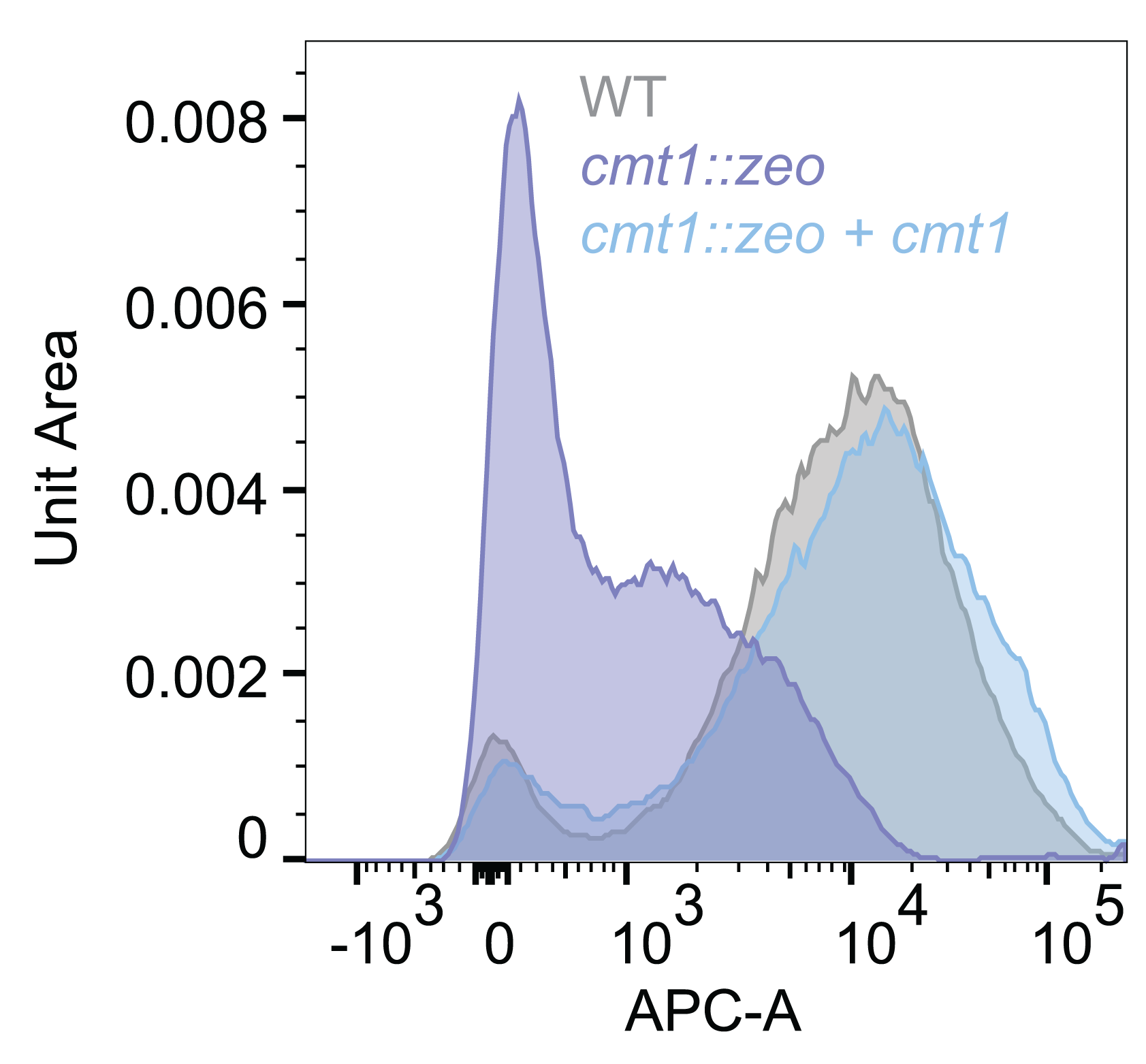

Supplement: S2 Fig — Cells were grown until mid-log and incubated with anti-His Alexa Fluor 647. Stained cells were then washed and analyzed by flow cytometry. Representative data scaled by unit area is shown as a histogram. The strains contain genome-integrated constructs at the attB2 site that are either an empty vector or the complement cmt1 allele induced with 1mM theophylline. (TIF) [file pgen.1011127.s002.tif]

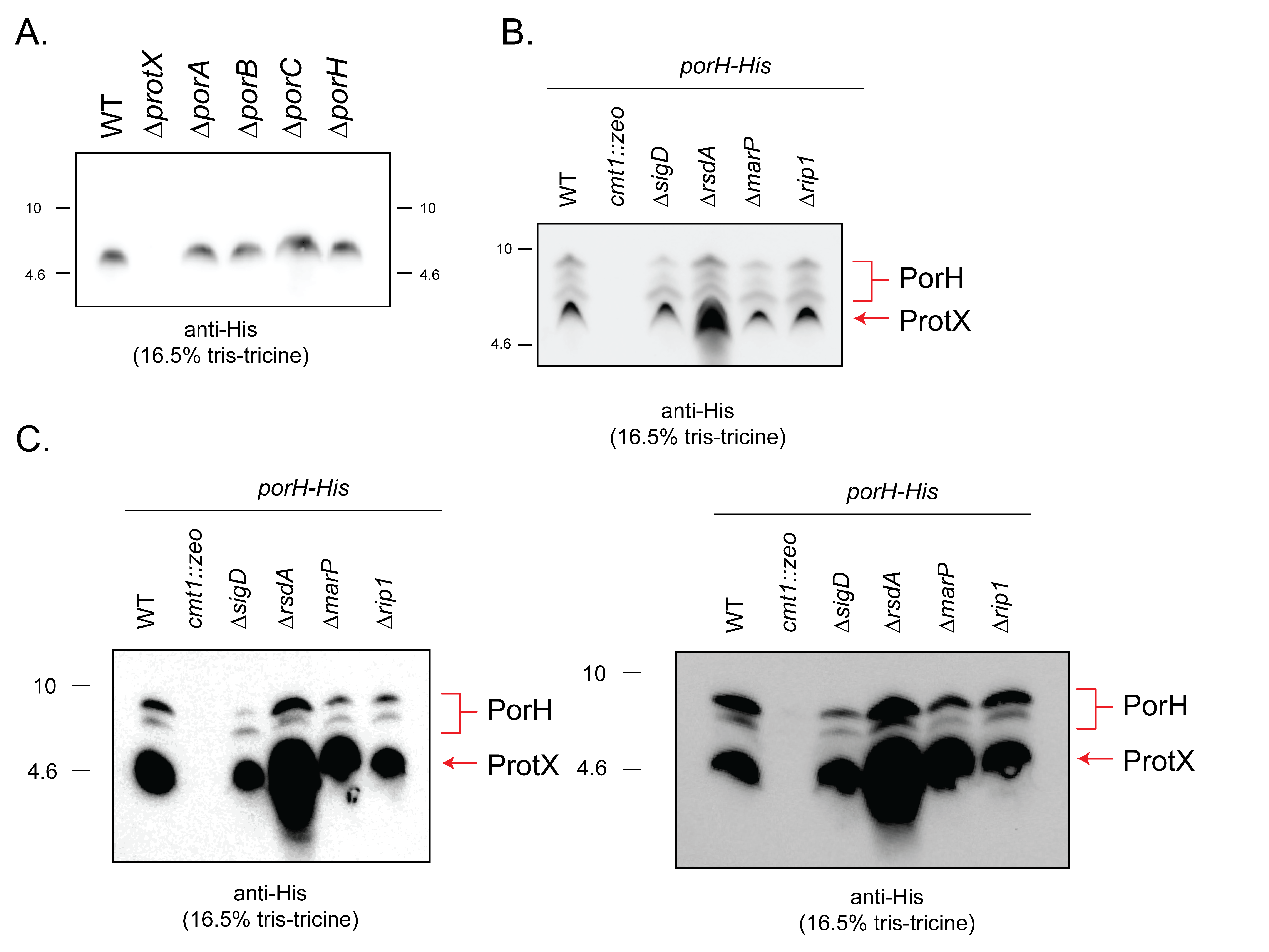

Supplement: S3 Fig — (A) The indicated strains without a His-tagged construct were analyzed by immunoblot analysis using a commercial anti-His antibody. Note that native ProtX (Cgp_2785) appears to be detected by the antibody as a non-specific band. (B) The indicated strains were analyzed by immunoblot. Red bracket and arrows indicate the PorH-His proteoforms and native ProtX, respectively. Note that ProtX is induced when the σD pathway is activated by deletion of rsdA. (C) Additional two replicates of the same experiment shown in (B). (TIF) [file pgen.1011127.s003.tif]

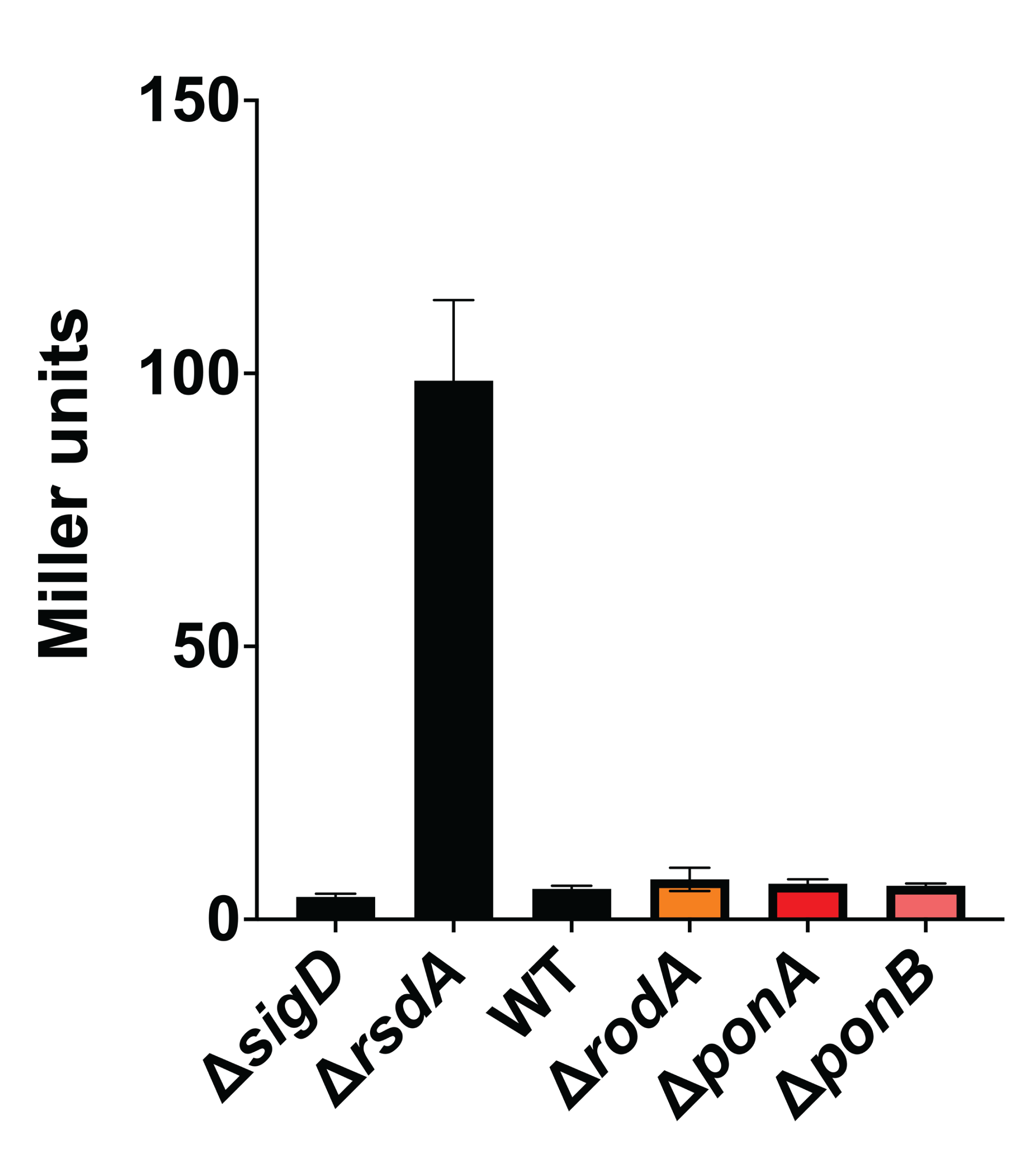

Supplement: S4 Fig — σD reporter activity in the indicated strains was measured by β-galactosidase activity. Measurements were made in triplicate and the error bars represent standard deviation. (TIF) [file pgen.1011127.s004.tif]

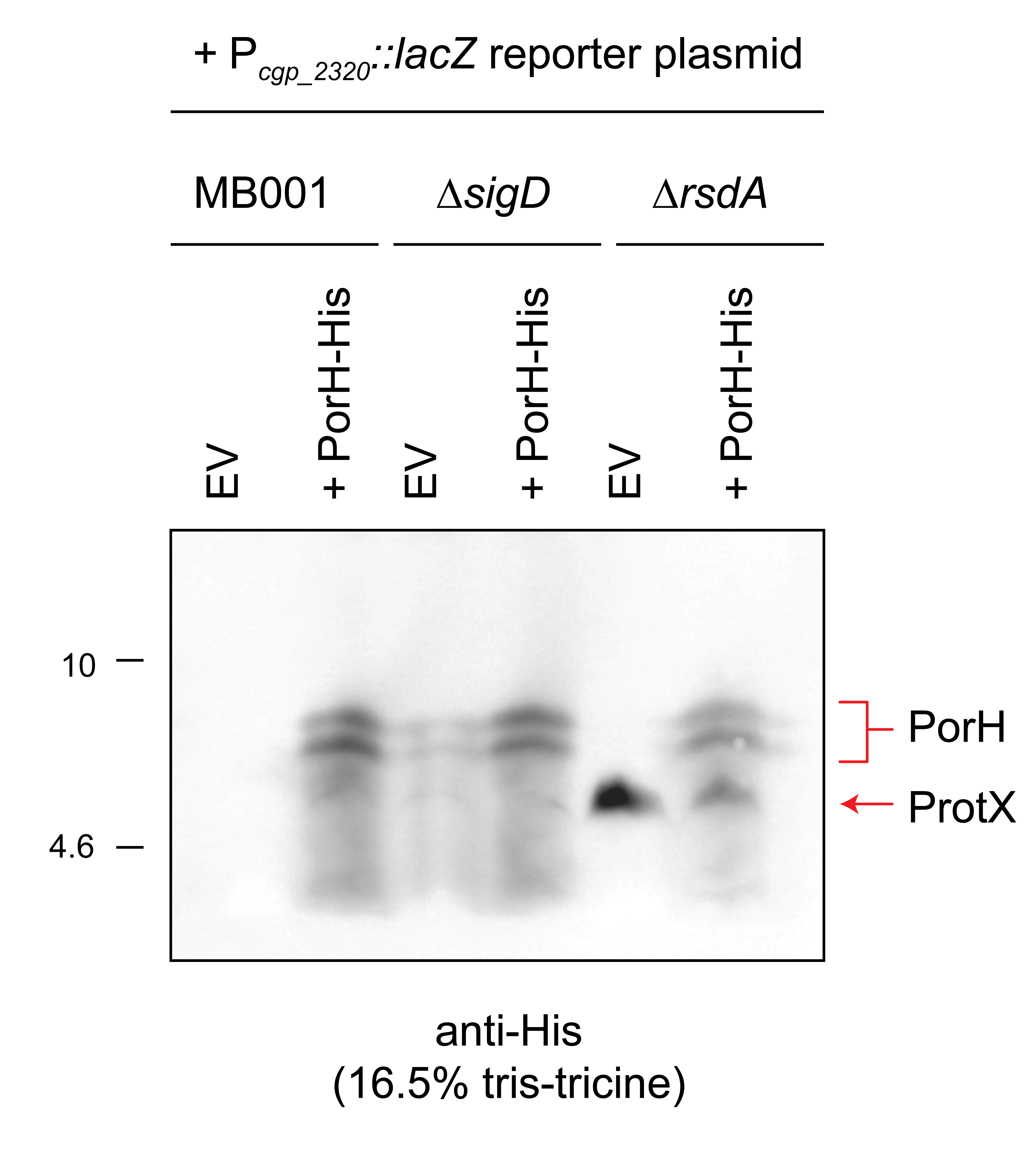

Supplement: S5 Fig — The indicated strains containing the PorH-His overexpression plasmid (pEMH306) or an empty vector (pEMH309) as well as the σD reporter plasmid (pEMH304) were analyzed by immunoblot analysis. (TIF) [file pgen.1011127.s005.tif]

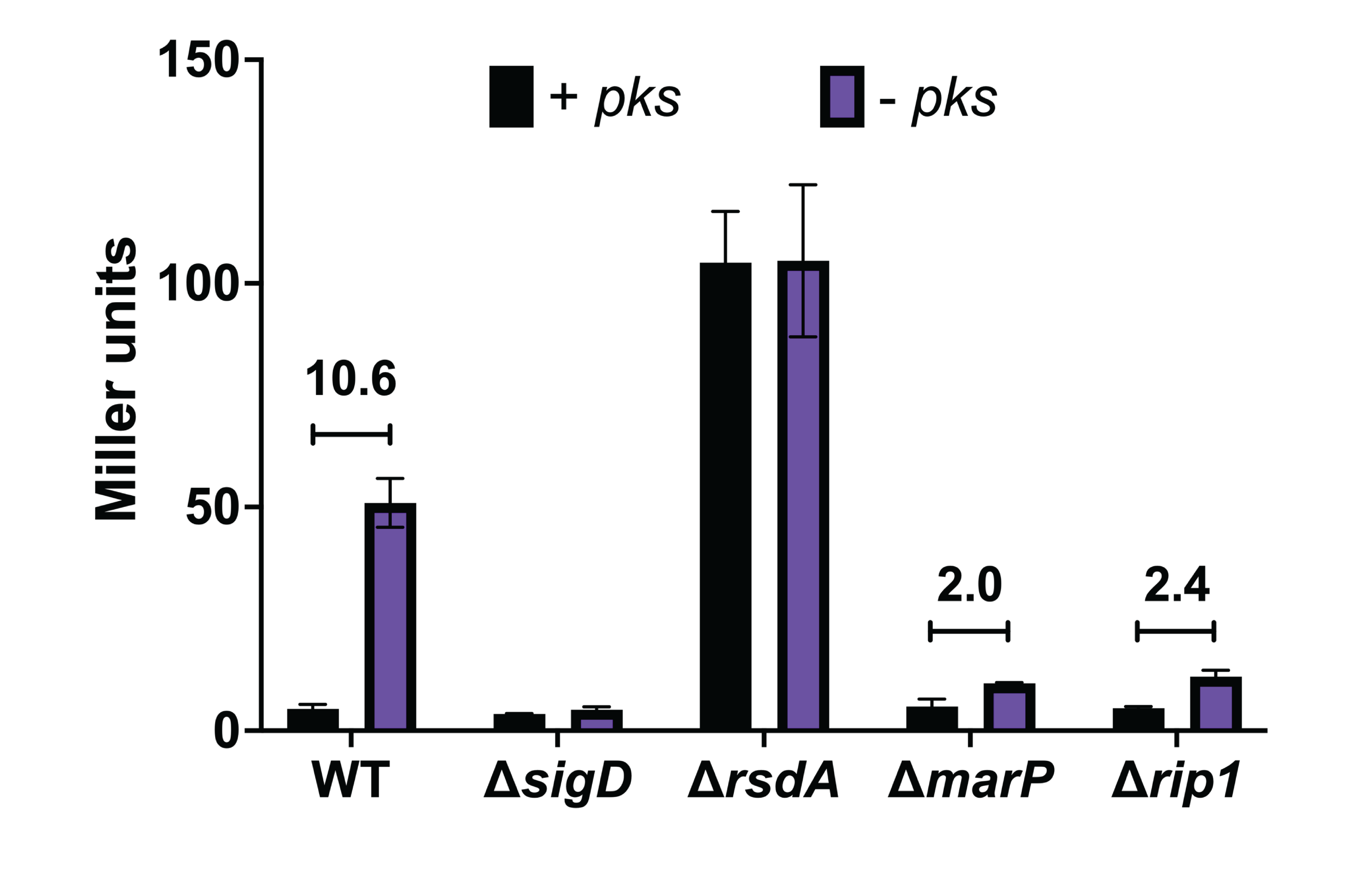

Supplement: S6 Fig — (A) Reporter activity of the listed genetic backgrounds expressing pks (black) or with pks disrupted (purple) was analyzed by β-galactosidase activity. The average of three replicates is displayed with error bars denoting standard deviation. The displayed numbers indicate the fold change in reporter activity between + pks and–pks stress condition of the listed genetic background. (TIF) [file pgen.1011127.s006.tif]

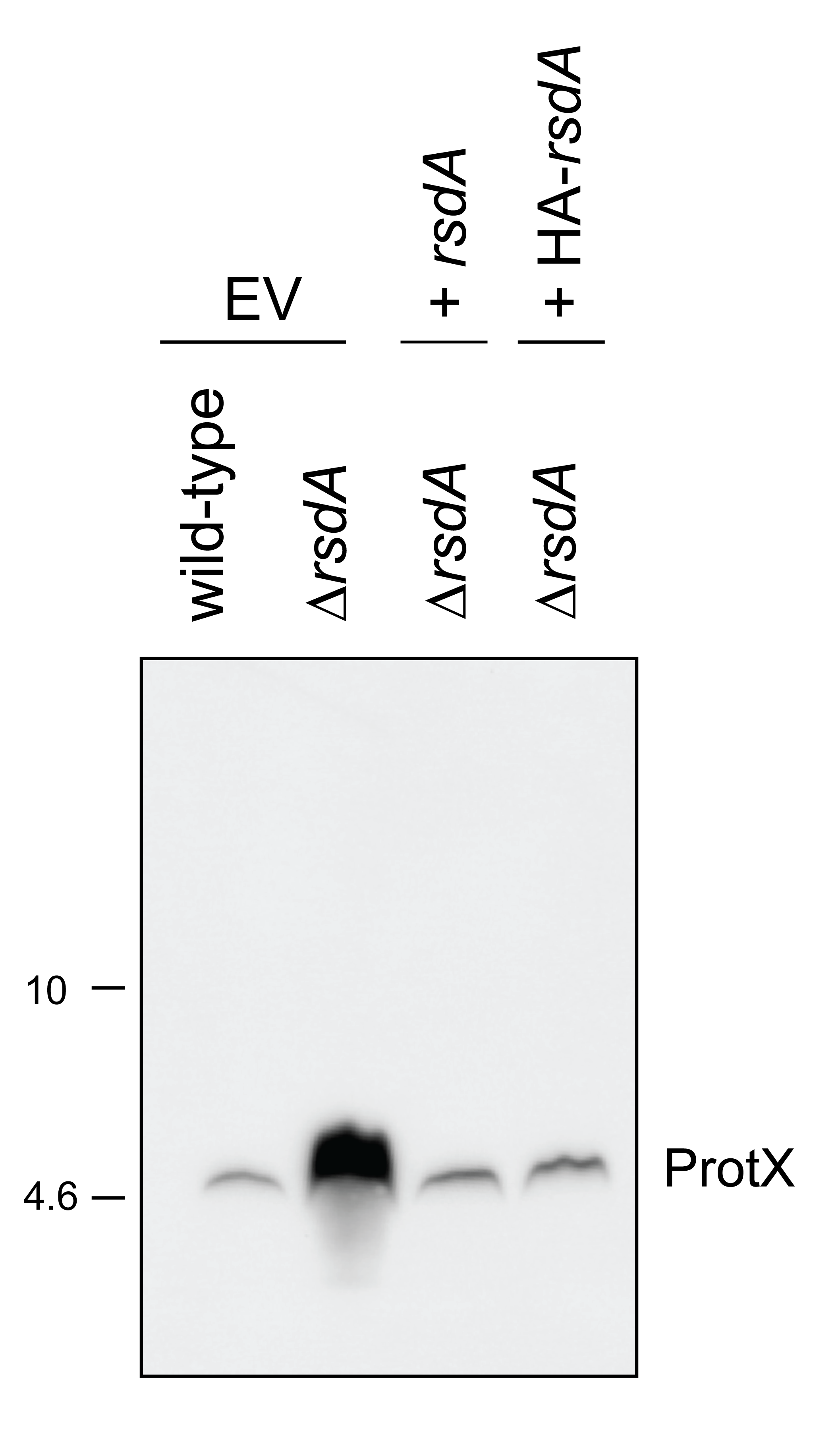

Supplement: S7 Fig — Immunoblot of the indicated genetic backgrounds harboring either an empty vector (EV), a multicopy plasmid expressing untagged RsdA, or a multicopy plasmid expressing HA-RsdA. The anti-His primary antibody detects native ProtX, the production of which is induced upon activation of the σD pathway (see S3 Fig). (TIF) [file pgen.1011127.s007.tif]

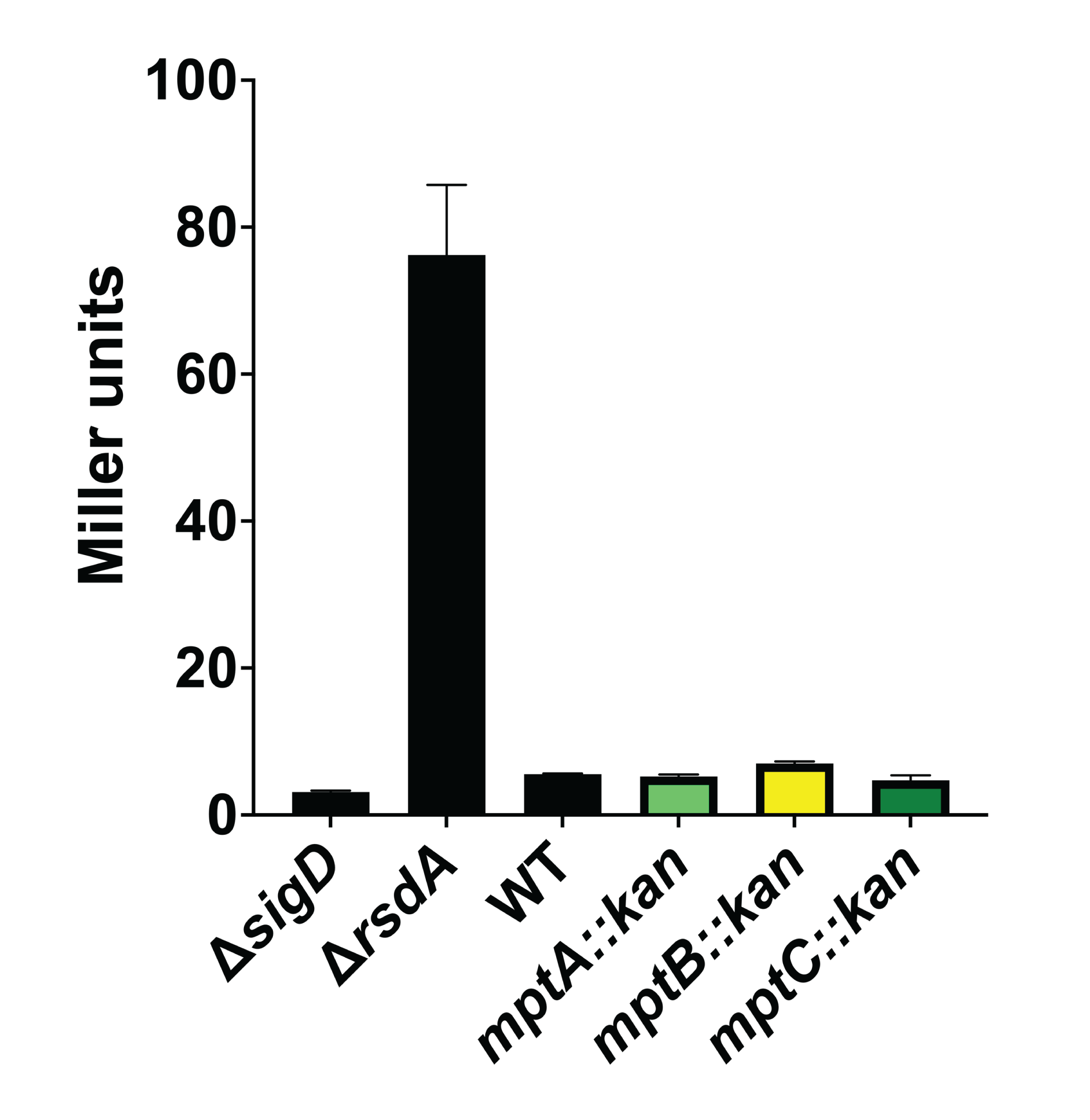

Supplement: S8 Fig — σD activity of controls and LM/LAM biosynthetic mutants was measured by β-galactosidase assay. Data shown are three biological replicates and error bars represent standard deviation. (TIF) [file pgen.1011127.s008.tif]

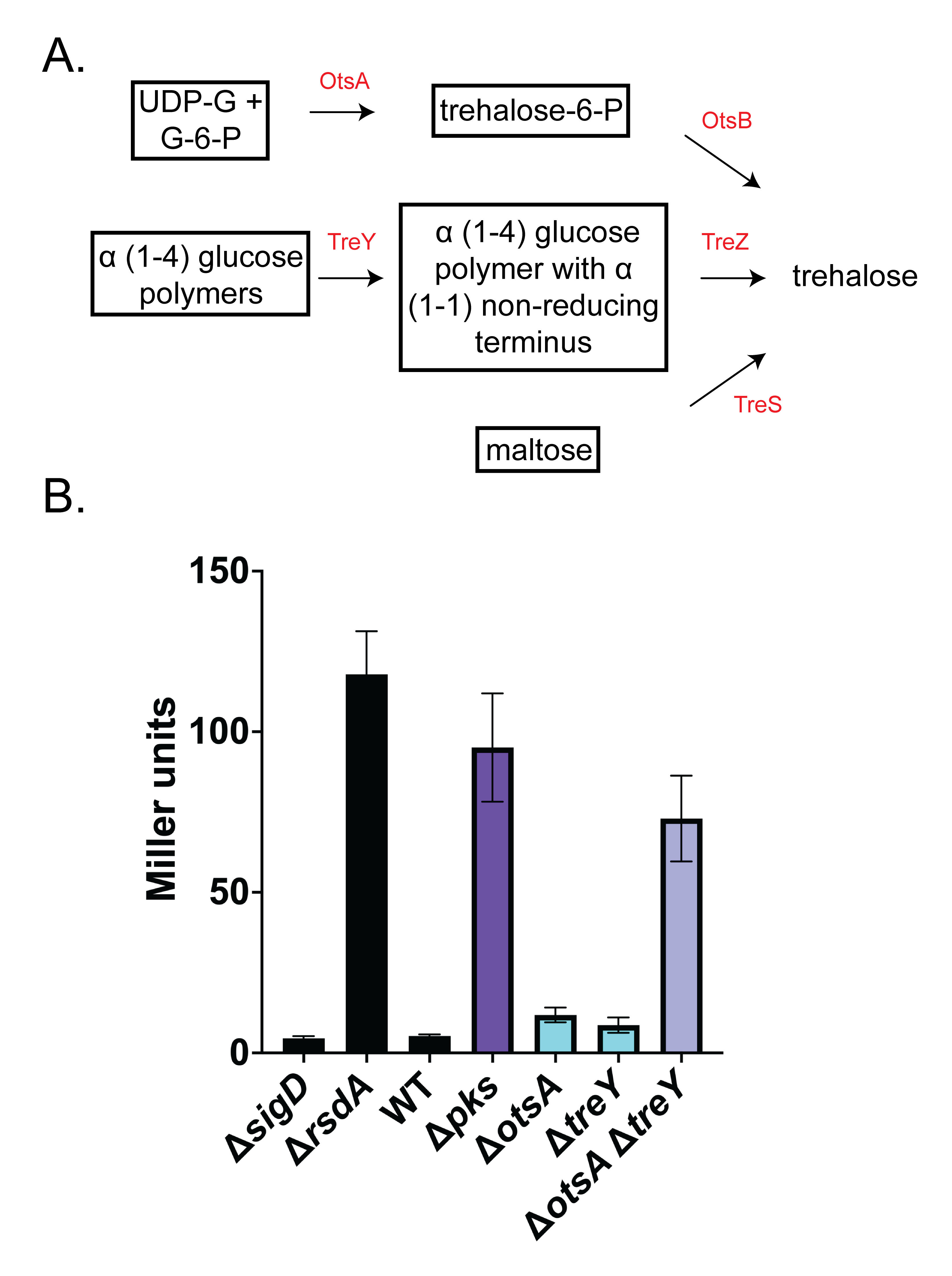

Supplement: S9 Fig — (A) Schematic of the three trehalose biosynthetic pathways of Cglu. Adapted from [62, 69]. (B) β-galactosidase assay of trehalose biosynthetic mutants and controls. σD activity of the listed cultures was measured in biological triplicate. Error bars represent standard deviation. (TIF) [file pgen.1011127.s009.tif]

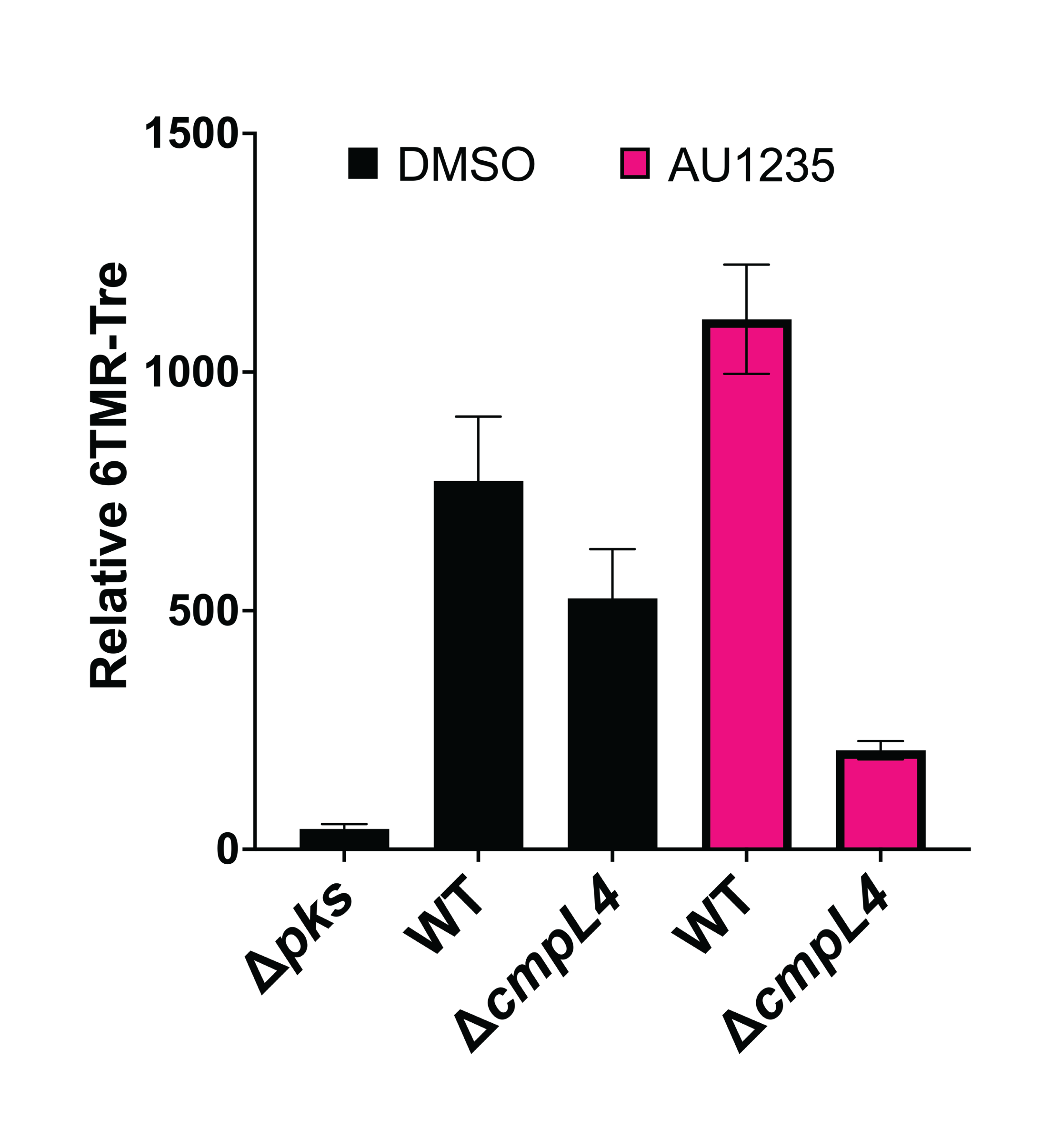

Supplement: S10 Fig — The listed strains were grown until mid-log with either the vehicle control (DMSO) or 0.025mM AU1235. Equivalent numbers of cells were stained with 100μM 6TMR-Tre for 30 minutes in biological triplicate. The OD600 and 6TMR-Tre incorporation were measured and used to calculate relative 6TMR-Tre staining (6TMR-Tre fluorescence emission/OD600). Error bars represent standard deviation. (TIF) [file pgen.1011127.s010.tif]
