# Supplementary material for: The conserved σD envelope stress response monitors multiple aspects of envelope integrity in corynebacteria
Source: PLoS Genet. 2024 Jun 3;20(6):e1011127. doi: 10.1371/journal.pgen.1011127 (PMC11175481; doi:10.1371/journal.pgen.1011127)
Supplement: S2 Table — (DOCX) [file pgen.1011127.s012.docx]

**Table S2: Strain list**

| **Strain number** | **Genotype** | **Strain construction** | **Source/**  **reference** |
| --- | --- | --- | --- |
| H60 | MB001 (ATCC 13032 ΔCGP1 (cg1507-gp1524) ΔCGP2 (cg1746-1752) ΔCGP3 (cg1890-cg2071) |  | (1) |
| H1111 | Δ*protX* | Deletion of *protX* (allelic exchange) | This study |
| H1241 | Δ*porH (cgp_3009)* | Deletion of *porH* (allelic exchange) | This study |
| H1248 | Δ*porH* pEMH25 | H1241/pEMH25 | This study |
| H1249 | Δ*porH* pEMH26 | H1241/pEMH26 | This study |
| H1448 | Δ*porH* pEMH27 | H1241/pEMH27 | This study |
| H1134 | *cmt1::zeo* | Disruption of *cmt1* with zeocin-resistance cassette driven by native *cmt1* promoter (allelic exchange) | This study |
| H1179 | MB001 pEMH27 | H60/pEMH27 | This study |
| H1195 | *cmt1::zeo* pEMH27 | H1134/pEMH27 | This study |
| H1236 | Tn library pEMH27 | Tn library/pEMH27 | This study |
| H1284 | Δ*sigD (cgp_0696)* | Deletion of *sigD* (allelic exchange) | This study |
| H1285 | Δ*rsdA (cgp_0697)* | Deletion of *rsdA* (allelic exchange) | This study |
| H952 | Δ*marP (cgp_0356)* | Deletion of *marP* (allelic exchange) | This study |
| H1286 | Δ*rip1 (cgp_2207)* | Deletion of *rip1* | This study |
| H1294 | Δ*sigD* pEMH27 | H1284/pEMH27 | This study |
| H1297 | Δ*rsdA* pEMH27 | H1285/pEMH27 | This study |
| H1253 | Δ*marP* pEMH27 | H952/pEMH27 | This study |
| H1300 | Δ*rip1* pEMH27 | H1286/pEMH27 | This study |
| H1329 | MB001 *attB2*(EV) | H60/pACM185 | This study |
| H1340 | MB001 *attB2*(EV) pEMH27 | H1329/pEMH27 | This study |
| H1365 | *cmt1::zeo* *attB2*(EV) | H1134/pACM185 | This study |
| H1399 | *cmt1::zeo* *attB2*(EV) pEMH27 | H1365/pEMH27 | This study |
| H1366 | *cmt1::zeo* *attB2*::*cmt1* | H1134/pACM64 | This study |
| H1400 | *cmt1::zeo* *attB2*::*cmt1* pEMH27 | H1366/pEMH27 | This study |
| H1409 | Δ*sigD attB2*(EV) | H1284/pACM185 | This study |
| H1306 | Δ*sigD attB2*(EV) pEMH27 | H1409/pEMH27 | This study |
| H1410 | Δ*sigD attB2::sigD* | H1284/pEMH73 | This study |
| H1350 | Δ*sigD attB2::sigD* pEMH27 | H1410/pEMH27 | This study |
| H1288 | Δ*sigD attB2::cmt1* | H1284/pACM64 | This study |
| H1309 | Δ*sigD attB2::cmt1* pEMH27 | H1288/pEMH27 | This study |
| H1411 | Δ*rsdA attB2*(EV) | H1297/pACM185 | This study |
| H1351 | Δ*rsdA attB2*(EV) pEMH27 | H1411/pEMH27 | This study |
| H1506 | MB001 pEMH121 | H60/pEMH121 | This study |
| H1507 | Δ*sigD* pEMH121 | H1294/pEMH121 | This study |
| H1508 | Δ*rsdA* pEMH121 | H1297/pEMH121 | This study |
| H1617 | Δ*marP* pEMH121 | H952/pEMH121 | This study |
| H1618 | Δ*rip1* pEMH121 | H1286/pEMH121 | This study |
| H1555 | *cmt1::zeo* pEMH121 | H1134/pEMH121 | This study |
| H2261 | Δ*pks (cgp_3178)* | Deletion of *pks* (allelic exchange) | (2) |
| H1541 | Δ*pks* pEMH121 | H2261/pEMH121 | This study |
| H2338 | MB001 pEMH304 | H60/pEMH304 | This study |
| H2362 | MB001 pEMH304 pEMH309 | H2338/pEMH309 | This study |
| H2386 | MB001 pEMH304 pEMH306 | H2338/pEMH306 | This study |
| H2371 | Δ*sigD* pEMH304 | H1294/pEMH304 | This study |
| H2434 | Δ*sigD* pEMH304 pEMH309 | H2371/pEMH309 | This study |
| H2387 | Δ*sigD* pEMH304 pEMH306 | H2371/pEMH306 | This study |
| H2341 | Δ*rsdA* pEMH304 | H1297/pEMH304 | This study |
| H2364 | Δ*rsdA* pEMH304 pEMH309 | H2364/pEMH309 | This study |
| H2388 | Δ*rsdA* pEMH304 pEMH306 | H2364/pEMH306 | This study |
| H793 | Δ*rodA* | Deletion of *rodA* (allelic exchange) | (3) |
| H2541 | Δ*rodA* pEMH121 | H793/pEMH121 | This study |
| H101 | Δ*ponA* | Deletion of *ponA* (allelic exchange) | (4) |
| H2542 | Δ*ponA* pEMH121 | H101/pEMH121 | This study |
| H517 | Δ*ponB* | Deletion of *ponB* (allelic exchange) | (3) |
| H2543 | Δ*ponB* pEMH121 | H517/pEMH121 | This study |
| H2093 | MB001 pEWL54 | H60/pEWL54 | This study |
| H3111 | MB001 pEMH121 pEWL103 | H1506/pEWL103 | This study |
| H3172 | *pks::kan* pEMH121 | Disruption of *pks* in H3111 (recombineering) | This study |
| H2564 | Δ*sigD* pEWL54 | H1284/pEWL54 | This study |
| H2608 | Δ*sigD pks::kan* | Disruption of *pks* in H2564 | This study |
| H3216 | Δ*sigD pks::kan* pEMH121 | H2608/pEMH121 | This study |
| H3158 | Δ*rsdA* pEMH121 pEWL103 | H1508/pEWL103 | This study |
| H3217 | Δ*rsdA pks::kan* pEMH121 | Disruption of *pks* in H3158 (recombineering) | This study |
| H2565 | Δ*marP* pEWL54 | H952/pEWL54 | This study |
| H2609 | Δ*marP pks::kan* | Disruption of *pks* in H2565 (recombineering) | This study |
| H3218 | Δ*marP pks::kan* pEMH121 | H2609/pEMH121 | This study |
| H2566 | Δ*rip1* pEWL54 | H1297/pEWL54 | This study |
| H2610 | Δ*rip1 pks::kan* | Disruption of *pks* in H2566 (recombineering) | This study |
| H3219 | Δ*rip1 pks::kan* pEMH121 | H2610/pEMH121 | This study |
| H1657 | Δ*rsdA* pEMH120 | H1297/pEMH120 | This study |
| H1756 | Δ*rsdA* pEMH193 | H1297/pEMH!93 | This study |
| H1738 | Δ*marP* Δ*rsdA* | Deletion of *rsdA* in H952 (allelic exchange) | This study |
| H1771 | Δ*marP* Δ*rsdA* pEMH193 | H1738/pEMH193 | This study |
| H1760 | Δ*rip1* Δ*rsdA* | Deletion of *rsdA* in H1286 (allelic exchange) | This study |
| H1781 | Δ*rip1* Δ*rsdA* pEMH193 | H1760/pEMH193 | This study |
| H1857 | Δ*marP* Δ*rip1* Δ*rsdA* | Deletion of *rip1* in H1738 (allelic exchange) | This study |
| H1904 | Δ*marP* Δ*rip1* Δ*rsdA* pEMH193 | H1857/pEMH193 | This study |
| H1697 | MB001 *attB2(Zeo)::pks* | H60/pEMH161 | This study |
| H1761 | Δ*pks attB2(Zeo)::pks* | Deletion of *pks* in H1697 (allelic exchange) | This study |
| H1785 | Δ*rsdA* Δ*pks attB2(Zeo)::pks* | Deletion of *rsdA* in H1761 (allelic exchange) | This study |
| H1909 | Δ*rsdA* Δ*pks attB2(Zeo)::pks* pEMH120 | H1785/pEMH120 | This study |
| H1910 | Δ*rsdA* Δ*pks attB2(Zeo)::pks* pEMH193 | H1785/pEMH193 | This study |
| H1842 | Δ*marP* Δ*rsdA* Δ*pks attB2(Zeo)::pks* | Deletion of *marP* in H1785 (allelic exchange) | This study |
| H2843 | Δ*marP* Δ*rsdA* Δ*pks attB2(Zeo)::pks* pEMH193 | H1842/pEMH!93 | This study |
| H1891 | Δ*rip1*Δ*rsdA* Δ*pks attB2(Zeo)::pks* | Deletion of *rip1* in H1785 (allelic exchange) | This study |
| H1925 | Δ*rip1*Δ*rsdA* Δ*pks attB2(Zeo)::pks* pEMH193 | H1891/pEMH193 | This study |
| H2653 | *cmpL1::kan (cgp_3174)* | Disruption of *cmpL1* in H2093 (recombineering) | This study |
| H2966 | *cmpL1::kan* pEMH121 | H2653/pEMH121 | This study |
| H1476 | Δ*cmpL4 (cgp_0284)* | Deletion of *cmpL4* (allelic exchange) | This study |
| H1976 | Δ*cmpL4* pEMH121 | H1476/pEMH121 | This study |
| H2573 | Δ*cmpL4* pEWL54 | H1476/pEWL54 | This study |
| H2659 | Δ*cmpL4 cmpL1::kan* | Disruption of *cmpL1*in H2573 (recombineering) | This study |
| H2720 | Δ*cmpL4 cmpL1::kan* pEMH121 | H2659/pEMH121 | This study |
| H2854 | Δ*cmpL4* Δ*cmpL3 (cgp_1054)* | Deletion of *cmpL3* in H2573 (recombineering) | This study |
| H2861 | Δ*cmpL4* Δ*cmpL3* pEWL54 | H2854/pEWL54 | This study |
| H2887 | Δ*cmpL4* Δ*cmpL3* Δ*cmpL2 (cgp_0623)* | Deletion of *cmpL2* in H2861 (recombineering) | This study |
| H2894 | Δ*cmpL4* Δ*cmpL3* Δ*cmpL2* pEWL54 | H2887/pEWL54 | This study |
| H2909 | Δ*cmpL2-4 cmpL1::kan (cgp_3174/cgp_0623/cgp_1054/cgp_3174)* | Disruption of *cmpL1* in H2894 (recombineering) | This study |
| H2177 | Δ*pccB* (*cgp_3177)* | Deletion of *pccB* (allelic exchange) | This study |
| H2228 | Δ*pccB* pEMH121 | H2177/pEMH121 | This study |
| H2206 | Δ*fadD2 (cgp_3179)* | Deletion of *fadD2* (allelic exchange) | This study |
| H2227 | Δ*fadD2* pEMH121 | H2206/pEMH121 | This study |
| H2678 | Δ*pptA* (*cgp_2171)* | Deletion of *pptA* in H2093 (recombineering) | This study |
| H2710 | Δ*pptA* pEMH121 | H2678/pEMH121 | This study |
| H2677 | Δ*cmrA (cgp_2717)* | Deletion of *cmrA* in H2093 (recombineering) | This study |
| H2705 | Δ*cmrA* pEMH121 | H2677/pEMH121 | This study |
| H2864 | Δ*mmpA (cgp_3165)* | Deletion of *mmpA* in H2093 (recombineering) | This study |
| H2871 | Δ*mmpA* pEMH121 | H2864/pEMH121 | This study |
| H2654 | Δ*tmaT (cgp_3163)* | Deletion of *tmaT* in H2093 (recombineering) | This study |
| H2676 | Δ*tmaT* pEMH121 | H2654/pEMH121 | This study |
| H2262 | Δ*ahfA (cgp_0475)* | Deletion of *ahfA* (allelic exchange) | (2) |
| H2076 | Δ*ahfA* pEMH121 | H2262/pEMH121 | This study |
| H1666 | Δ*otsA (cgp_2907)* | Deletion of *otsA* (allelic exchange) | (2) |
| H1554 | Δ*otsA* pEMH121 | H1666/pEMH121 | This study |
| H2508 | Δ*treY (cgp_2323)* | Deletion of *treY* (allelic exchange) | This study |
| H2527 | Δ*treY* pEMH121 | H2508/pEMH121 | This study |
| H2536 | Δ*otsA* Δ*treY* | Deletion of *otsA* in H2508 (allelic exchange) | This study |
| H2544 | Δ*otsA* Δ*treY* pEMH121 | H2536/pEMH121 | This study |
| H3234 | *ubiA::kan* pEMH121 | Disruption of *ubiA* in H3111 (recombineering) | This study |
| H3155 | *emb::kan* pEMH121 | Disruption of *emb* in H3111 (recombineering) | This study |
| H3275 | *aftA::kan* pEMH121 | Disruption of *aftA* in H3111 (recombineering) | This study |
| H3174 | *aftB::kan* pEMH121 | Disruption of *aftB* in H3111 (recombineering) | This study |
| H2657 | Δ*aftD (cgp_3161)* | Deletion of *aftD* in H2093 (recombineering) | This study |
| H2672 | Δ*aftD* pEMH121 | H2657/pEMH121 | This study |
| H2656 | Δ*aftC (cgp_2077)* | Deletion of *aftC* in H2093 (recombineering) | This study |
| H2673 | Δ*aftC* pEMH121 | H2656/pEMH121 | This study |
| H2438 | *mptA::kan* | Disruption of *mptA* in H2093 (recombineering) | This study |
| H2459 | *mptA::kan* pEMH121 | H2438/pEMH121 | This study |
| H2439 | *mptB::kan* | Disruption of *mptB* in H2093 (recombineering) | This study |
| H2460 | *mptB::kan* pEMH121 | H2439/pEMH121 | This study |
| H2440 | *mptC::kan* | Disruption of *mptC* in H2093 (recombineering) | This study |
| H2461 | *mptC::kan* pEMH121 | H2440/pEMH121 | This study |

**Supplemental bibliography**

1. Baumgart M, Unthan S, Rückert C, Sivalingam J, Grünberger A, Kalinowski J, Bott M, Noack S, Frunzke J. 2013. Construction of a prophage-free variant of *Corynebacterium glutamicum* ATCC 13032 for use as a platform strain for basic research and industrial biotechnology. *Appl Environ Microbiol* 79:6006–6015.

2. McKitterick AC, Bernhardt TG. 2022. Phage resistance profiling identifies new genes required for biogenesis and modification of the corynebacterial cell envelope. *Elife* 11:e79981.

3. Sher JW, Lim HC, Bernhardt TG. 2021. Polar Growth in *Corynebacterium glutamicum* Has a Flexible Cell Wall Synthase Requirement. *mBio* 12:10.1128/mbio.00682-21.

4. Sher JW, Lim HC, Bernhardt TG. 2020. Global phenotypic profiling identifies a conserved actinobacterial cofactor for a bifunctional PBP-type cell wall synthase. Elife 9.
