# Supplementary material for: The conserved σD envelope stress response monitors multiple aspects of envelope integrity in corynebacteria
Source: PLoS Genet. 2024 Jun 3;20(6):e1011127. doi: 10.1371/journal.pgen.1011127 (PMC11175481; doi:10.1371/journal.pgen.1011127)
Supplement: S3 Table — (DOCX) [file pgen.1011127.s013.docx]

**Table S3: Plasmids used in this study**

| **Plasmid name** | **Information** | **Source** |
| --- | --- | --- |
| pCRD206 | Kan^R^*, sacB* counterselection, temperature-sensitive origin | (1) |
| pEMH1 | pCRD206::*protX* | This study |
| pEMH5 | pCRD206::*cmt1-zeo* | This study |
| pEMH19 | pCRD206::*porH* | This study |
| pEMH54 | pCRD206::*rsdA* | This study |
| pEMH53 | pCRD206::*sigD* | This study |
| pEMH55 | pCRD206::*rip1* | This study |
| pJWS115 | pCRD206::*marP* | This study |
| pEMH105 | pCRD206::*cmpL4* | This study |
| pEMH135 | pCRD206::*pccB* | This study |
| pEMH134 | pCRD206::*fadD2* | This study |
| pACM163 | pCRD206::*pks* | (2) |
| pEMH361 | pCRD206::*treY* | This study |
| pACM30 | pCRD206::*otsA* | (2) |
| pEWL85 | pCRD206(Kan^R^)::P*_sod_*-*cre* | This study |
| pEWL89 | pCRD206(Apra^R^)::P*_sod_*-*cre* | This study |
| pEWL103 | pCRD206(Apra^R^)::P*_tac_* riboE1-SSAP/SSB | This study |
| pEWL74 | pHC632 derivative, contains LoxP71-Kan^R^-LoxP66 region in MCS | This study |
| pK-PIM | Integrating vector | (3) |
| pACM185 | P*_sod_* riboE1 empty vector (Kan^R^, pK-PIM derivative, theophylline inducible) | (2) |
| pACM64 | P*_sod_* riboE1(Kan^R^)::*cmt1* | (2) |
| pACM186 | P*_sod_* riboE1(Kan^R^)::*pks* | (2) |
| pEMH161 | P*_sod_* riboE1(Zeo^R)^::*pks* | This study |
| pEMH73 | P*_sod_* riboE1(Kan^R^)::*sigD* | This study |
| pEMH169 | P*_sod_* riboE1(Kan^R^)::HA-*rsdA* | This study |
| pTGR5 | P*_tac::_gfp* (Kan^R^, P_TAC_-eGFP, pGA1 mini replicon) | (4) |
| pAM246 | P*_tac::_gfp* (Cam^R^, P_TAC_-eGFP, pGA1 mini replicon) | This study |
| pEWL54 | pTGR5(Cam^R^)::P*_tac_*-SSAP/SSB | This study |
| pEWL65 | pHC632 variant, contains LoxP66/LoxP71 sites | This study |
| pEWL68 | pTGR5(Cam^R^):: P*_tac_*-SSAP/SSB, with pEC-XK99E origin | This study |
| pEWL69 | pTGR5(Cam^R^):: P*_sod_*-*cre*, with pEC-XK99E origin | This study |
| pEWL73 | pTGR5(Cam^R^):: P*_sod_*-*cre*, with pEC-XK99E origin, optimized 5’-UTR/RBS | This study |
| pEMH119 | pACM246 with native BamHI site deleted | This study |
| pEMH120 | P*_tac_* empty vector (pACM246-derived), *eGFP* deleted, insertion of BamHI site in MCS | This study |
| pEMH193 | pEMH120::*HA-rsdA* | This study |
| pEMH3 | P*_sod_* empty vector (Kan^R^, pGA1 mini replicon, constitutive expression) | This study |
| pEMH6 | P*_sod_* empty vector (Cam^R^, pGA1 mini replicon, constitutive expression) | This study |
| pEMH17 | pEMH6::*porH* | This study |
| pEMH25 | P*_sod_* empty vector (Cam^R^, pGA1 mini replicon, constitutive expression, native 6x His deleted) | This study |
| pEMH90 | P*_cgp_2320_*::*mScarlet* | This study |
| pEMH121 | P*_cgp_2320_*::*lacZ* | This study |
| pEMH304 | P*_cgp_2320_*::*lacZ* (Kan^R^) | This study |
| pEMH26 | pEMH117, deletion of native 6x His to match pEMH25 | This study |
| pEMH27 | pEMH25::*porH-His* | This study |
| pFSC | pXMJ19 (P*_tac_*::*cas9*, Cam^R^) | (5) |
| pEMH309 | pFSC empty vector, pFSC was digested with HindIII/EcoRI, blunted with NEB blunting kit, and ligated with T4 DNA ligase (NEB) | This study |
| pEMH306 | pFSC::*porH-His* | This study |

**BIBLIOGRAPHY**

1. Okibe N, Suzuki N, Inui M, Yukawa H. 2011. Efficient markerless gene replacement in *Corynebacterium glutamicum* using a new temperature-sensitive plasmid. *Journal of Microbiological Methods* 85:155–163.

2. McKitterick AC, Bernhardt TG. 2022. Phage resistance profiling identifies new genes required for biogenesis and modification of the corynebacterial cell envelope. *Elife* 11:e79981.

3. Oram M, Woolston JE, Jacobson AD, Holmes RK, Oram DM. 2007. Bacteriophage-based Vectors for Site-specific Insertion of DNA in the Chromosome of *Corynebacteria. Gene* 391:53–62.

4. Sher JW, Lim HC, Bernhardt TG. 2020. Global phenotypic profiling identifies a conserved actinobacterial cofactor for a bifunctional PBP-type cell wall synthase. *Elife* 9.

5. Ravasi P, Peiru S, Gramajo H, Menzella HG. 2012. Design and testing of a synthetic biology framework for genetic engineering of *Corynebacterium glutamicum. Microb Cell Fact* 11:147.
