# Supplementary material for: The conserved σD envelope stress response monitors multiple aspects of envelope integrity in corynebacteria
Source: PLoS Genet. 2024 Jun 3;20(6):e1011127. doi: 10.1371/journal.pgen.1011127 (PMC11175481; doi:10.1371/journal.pgen.1011127)
Supplement: S4 Table — (DOCX) [file pgen.1011127.s014.docx]

**Table S4: Oligonucleotides used in this study**

| **Primer name** | **Sequence (5’ to 3’)** | **Description (associated plasmid)** |
| --- | --- | --- |
| BH1 | AGTCGACCTGCAGGCATG | Forward, to amplify pCRD206 backbone |
| BH2 | ATCCAACAGGGACACCAG | Reverse, to amplify pCRD206 backbone |
| BH3 | TCCTGGTGTCCCTGTTGGATCTGCCATGATCGAGCGCG | Forward, to amplify upstream region of *protX* (pEMH1) |
| BH4 | AAGCGGTAACAGAGGTCATTGTGATCTCTCTTTCTG | Reverse, to amplify upstream region of *protX* (pEMH1) |
| BH5 | AATGACCTCTGTTACCGCTTCTTCCTAGTCG | Forward, to amplify downstream region of *protX* (pEMH1) |
| BH6 | TGCATGCCTGCAGGTCGACTTGGTGGCTGGGGTAAAGATTG | Reverse, to amplify downstream region of *protX* (pEMH1) |
| BH7 | GCAGAATAAATAAATCCTGGTGTCCC | Forward, diagnostic primer for pCRD206 derived vectors |
| BH8 | GGGTAACGCCAGGGTTTTCC | Reverse, diagnostic primer for pCRD206 derived vectors |
| BH53 | TCCTGGTGTCCCTGTTGGATTGCAATTCCAAGAATGGATG  TCAACTTGGCAAGCTTCATACGTCTTTC | Forward, to amplify upstream region of *cmt1* (pEMH5) |
| BH54 | TCAACTTGGCAAGCTTCATACGTCTTTC | Reverse, to amplify upstream region of *cmt1* (pEMH5) |
| BH55 | TATGAAGCTTGCCAAGTTGACCAGTGCC | Forward, to amplify zeocin-resistance cassette (pEMH5) |
| BH79 | CTAGCTCAAAGTCCTGCTCCTCGGCCAC | Reverse, to amplify zeocin-resistance cassette (pEMH5) |
| BH58 | TGCATGCCTGCAGGTCGACTTCTGCATTGTCGATATTCC | Forward, to amplify downstream region of *cmt1* (pEMH5) |
| BH80 | GGAGCAGGACTTTGAGCTAGAGGCCTAG | Reverse, to amplify downstream region of *cmt1* (pEMH5) |
| BH81 | CAGAGATTTTTGGCTCGTTA | Forward, diagnostic primer for *cmt1* |
| BH82 | GTGACTGTCGCAGCAAG | Reverse, diagnostic primer for *cmt1* |
| BH45 | TCCTGGTGTCCCTGTTGGATGGCGAGTACGTCGACCTC | Forward, to amplify upstream region of *porH* (pEMH19) |
| BH46 | AGAAGTTATCAAGATCCATGAGAAATCTCCTTGAG | Reverse, to amplify upstream region of *porH* (pEMH19) |
| BH47 | CATGGATCTTGATAACTTCTCTTCCTAAGAG | Forward, to amplify downstream region of *porH* (pEMH19) |
| BH47 | TGCATGCCTGCAGGTCGACTTGAATATAGCGCTGGAAG | Reverse, to amplify downstream region of *porH* (pEMH19) |
| BH65 | TAACATTTCTGCAGGTCAAG | Forward, diagnostic primer for *porH* |
| BH66 | TCAGCAACTGCGCCA | Reverse, diagnostic primer for *porH* |
| BH216 | TCCTGGTGTCCCTGTTGGATGGGATGGGTGTTATCTGTC | Forward, to amplify upstream region of *rsdA* (pEMH54) |
| BH217 | TCGAGTCATTACTTGTTCTC | Reverse, to amplify upstream region of *rsdA* (pEMH54) |
| BH218 | GAGAACAAGTAATGACTCGAAGACTCCTGGAAACTAGG | Forward, to amplify downstream region of *rsdA* (pEMH54) |
| BH219 | TGCATGCCTGCAGGTCGACTGGTGTTAAGGCGAATATTAC | Reverse, to amplify downstream region of *rsdA* (pEMH54) |
| BH220 | TATTGTCAGCTACTTTTTATAGCTT | Forward, diagnostic primer for *rsdA* |
| BH221 | ACACAGATCATCCACTTCTTG | Reverse, diagnostic primer for *rsdA* |
| BH204 | TCCTGGTGTCCCTGTTGGATTCTGCTCTTTTAAACTGCTACTG | Forward, to amplify upstream region of *sigD* (pEMH53) |
| BH205 | TCTCCTGCTGATCAGCCAAGTTCTCGCAC | Reverse, to amplify upstream region of *sigD* (pEMH53) |
| BH206 | CTTGGCTGATCAGCAGGAGAACAAGTAATGACTCGAC | Forward, to amplify downstream region of *sigD* (pEMH53) |
| BH207 | TGCATGCCTGCAGGTCGACTCCTCCGACGGCGCCTTTC | Reverse, to amplify downstream region of *sigD* (pEMH53) |
| BH208 | CATTCCGCAGTAGTGAATGCAACC | Forward, diagnostic primer for *sigD* |
| BH209 | AGTCCAGAGTCACCATCATTGCCG | Reverse, diagnostic primer for *sigD* |
| BH210 | TCCTGGTGTCCCTGTTGGATATTCGGAGCACTCTGCCATGG | Forward, to amplify upstream region of *rip1* (pEMH55) |
| BH211 | AGAGTCGGATGGCTGCCACGAGGCGGAA | Reverse, to amplify upstream region of *rip1* (pEMH55) |
| BH212 | CGTGGCAGCCATCCGACTCTTTGGCTAACG | Forward, to amplify downstream region of *rip1* (pEMH55) |
| BH213 | TGCATGCCTGCAGGTCGACTAGGCCTCCACCATGAGTAC | Reverse, to amplify downstream region of *rip1* (pEMH55) |
| BH214 | CAGGCTGCCGCAGTAATTTCGAA | Forward, diagnostic primer for *rip1* |
| BH215 | CGGCCACATGATGGGCAGG | Reverse, diagnostic primer for *rip1* |
| J105 | ATAAATCCTGGTGTCCCTGTTGGATGGATGCACCGACTCTTAGACCGC | Forward, to amplify upstream region of *marP* (pJWS115) |
| J106 | CGACGACTAAGAAACCGCGCACAGGCTGGGGCTCAACAATGG | Reverse, to amplify upstream region of *marP* (pJWS115) |
| J107 | CCATTGTTGAGCCCCAGCCTGTGCGCGGTTTCTTAGTCGTCG | Forward, to amplify downstream region of *marP* (pJWS115) |
| J108 | CAAGCTTGCATGCCTGCAGGTCGACTGGCTGCTCAGAACCAACACTCG | Reverse, to amplify downstream region of *marP* (pJWS115) |
| J109 | GAGACAGTGCTCCTCCGCAAA | Reverse, diagnostic primer for *marP* |
| BH362 | TCCTGGTGTCCCTGTTGGATAAACGCCGGTGAAAAGGTAAAGG | Forward, to amplify upstream region of *cmpL4* (pEMH105) |
| BH363 | CAGCCTGCTTTTTCGCCACGGGGTTGCT | Reverse, to amplify upstream region of *cmpL4* (pEMH105) |
| BH364 | CGTGGCGAAAAAGCAGGCTGCACGTTAG | Forward, to amplify downstream region of *cmpL4* (pEMH105) |
| BH365 | TGCATGCCTGCAGGTCGACTGATCCAGGTCCAACAGGC | Reverse, to amplify downstream region of *cmpL4* (pEMH105) |
| BH366 | TAAGCAGTTTGTAGCTGCTTAAGAG | Forward, diagnostic primer for *cmpL4* |
| BH367 | CTGCATGGAGACTTCTGGGG | Reverse, diagnostic primer for *cmpL4* |
| BH536 | TCCTGGTGTCCCTGTTGGATTCGTCTGCCTGAAGATGTC | Forward, to amplify upstream region of *pccB* (pEMH135) |
| BH537 | GCATGATCGTGTTACTCAACTTCCGTCAG | Reverse, to amplify upstream region of *pccB* (pEMH135) |
| BH538 | GTTGAGTAACACGATCATGCCGATGTAAC | Forward, to amplify downstream region of *pccB* (pEMH135) |
| BH539 | TGCATGCCTGCAGGTCGACTTCTATTTCACCCCCCAGG | Reverse, to amplify downstream region of *pccB* (pEMH135) |
| BH540 | TCGATGTCGATACTGCAACCG | Forward, diagnostic primer for *pccB* |
| BH541 | GGCACTGCCCCGATGTTT | Reverse, diagnostic primer for *pccB* |
| BH542 | TCCTGGTGTCCCTGTTGGATAAGGCACCGCGAAGATGAATG | Forward, to amplify upstream region of *fadD2* (pEMH134) |
| BH543 | CTTGTTCCTGTAAATCCATGTGAACCCCTCTTAC | Reverse, to amplify upstream region of *fadD2* (pEMH134) |
| BH544 | CATGGATTTACAGGAACAAGCTAACTAGTTC | Forward, to amplify downstream region of *fadD2* (pEMH134) |
| BH545 | TGCATGCCTGCAGGTCGACTACATATCCTCCAGGCTCTC | Reverse, to amplify downstream region of *fadD2* (pEMH134) |
| BH546 | GAACCACCGGTTCAACCTGATTG | Forward, diagnostic primer for *fadD2* |
| BH547 | GCGATGCGAGCGTACTCCAAA | Reverse, diagnostic primer for *fadD2* |
| BH886 | TCCTGGTGTCCCTGTTGGATAGCCAACTTTTCAAGTCC | Forward, to amplify upstream region of *treY* (pEMH361) |
| BH887 | CACTATCGGGACGTGCCATGATGAAACAC | Reverse, to amplify upstream region of *treY* (pEMH361) |
| BH888 | CATGGCACGTCCCGATAGTGAGTTTTGATCC | Forward, to amplify downstream region of *treY* (pEMH361) |
| BH889 | TGCATGCCTGCAGGTCGACTTTGCCAATGAGCATGGCAAG | Reverse, to amplify downstream region of *treY* (pEMH361) |
| BH890 | TATGAAAAGGGTGCGCTGTGG | Forward, diagnostic primer for *treY* |
| BH891 | TTTGGCGTTGAGTGGTTGGGA | Reverse, diagnostic primer for *treY* |
| BH171 | TCAGAATTGGTTAAAAAGGATCTAGG | Forward, to amplify pACM186 vector (pEMH161) |
| BH172 | CGCACAGATGCGTAAGGAG | Reverse, to amplify pACM186 vector (pEMH161) |
| BH173 | TCTCCTTACGCATCTGTGCGGTACCTCTATCTGGTGCCCTAAAC | Forward, to amplify zeocin-resistance cassette (pEMH161) |
| BH174 | TCCTTTTTAACCAATTCTGATCAGTCCTGCTCCTCGGC | Reverse, to amplify zeocin-resistance cassette (pEMH161) |
| BH102 | GCACGTGAATTCACTGGC | Forward, to linearize pK-PIM derived vector |
| BH103 | CATCTTGTTGTTACCTCCTTTAG | Reverse, to linearize pK-PIM derived vector |
| J110 | TTGCGAAGCGCCATCTGACG | Forward, diagnostic primer for pK-PIM derived vectors |
| H296 | CGATTAAGTTGGGTAACGCCAG | Reverse, diagnostic primer for pK-PIM derived vectors |
| BH273 | AAGGAGGTAACAACAAGATGGCTGATACTGAGCGCGAG | Forward, to amplify *sigD* locus (-ATG) (pEMH73) |
| BH174 | CGGCCAGTGAATTCACGTGCTTACTTGTTCTCCTGCTGC | Reverse, to amplify *sigD* locus (-ATG) (pEMH73) |
| pEC-XK99 F | GCGGGACTCTGGGGTTCG | Forward, to linearize pTGR5 derivatives to switch out resistance cassette (pACM246/pEMH6) |
| pEC-XK99 R | GCGAAACGATCCTCATCCTGTCT | Reverse, to linearize pTGR5 derivatives to switch out resistance cassette (pACM246/pEMH6) |
| Cat F | AGACAGGATGAGGATCGTTTCGCATGGAGAAAAAAATCACTGGATATACCACC | Forward, to amplify CatR cassette (pACM246/pEMH6) |
| Cat R | CGAACCCCAGAGTCCCGCTTACGCCCCGCCCTGCC | Reverse, to amplify CatR cassette (pACM246/pEMH6) |
| EWL115 | CTAAGCTTCACCACCACC | Forward, to linearize pTGR5 derivative for insertion of SSAP/SSB gBlock (pEWL54) |
| EWL116 | TGTAAAAAATCCTTTCGCTAG | Reverse, to linearize pTGR5 derivative for insertion of SSAP/SSB gBlock (pEWL54) |
| EWL308 | CGTGAAGTTACCATCACGGA | Forward, to linearize pHC632 for insertion of LoxP66 and LoxP71 gBlocks (pEWL65) |
| EWL309 | GAATCAGACAATTGACGGCT | Forward, to linearize pHC632 for insertion of LoxP66 and LoxP71 gBlocks (pEWL65) |
| EWL316 | TTCGAGCTCCTGCAGGGTACCGCGGAACCCCTATTTGTTT | Forward, to amplify kanamycin resistance cassette from pK-PIM vector (pEWL74) |
| EWL317 | TATTCTAGAAGATCTGGATCCGCGCAGAAAAAAAGGATCT | Reverse, to amplify kanamycin resistance cassette from pK-PIM vector (pEWL74) |
| EWL335 | CAGGATGAGGATCGTTTCGC | Forward, to linearize pEWL54 (pEWL68) |
| ACM119 | CGAAGCCGCACGTCATCTAG | Reverse, to linearize pEWL54 (pEWL68) |
| EWL336 | GCGAAACGATCCTCATCCTG | Forward, to amplify origin from pEC-XK99E (pEWL68) |
| EWL337 | CTAGATGACGTGCGGCTTCG | Reverse, to amplify origin from pEC-XK99E (pEWL68) |
| H532 | CGCACAGATGCGTAAGGAGAAAATACC | Forward, to linearize pEWL68 (pEWL69) |
| ACM130 | TGGTCCCACCTGACCCCATG | Reverse, to linearize pEWL68 (pEWL69) |
| EWL318 | TCTCCTTACGCATCTGTGCGAAGCGCCTCATCAGCGGTAA | Forward, to amplify *cre* gBlock (pEWL69) |
| EWL328 | CATGGGGTCAGGTGGGACCATTAGTCACCATCCTCCAACAACC | Reverse, to amplify *cre* gBlock (pEWL69) |
| EWL346 | CTAGCGTTTCCCAAAGGATTTTTAATGTCTAATCTTCTGACCGTCCA | Forward, to linearize pEWL69 (pEWL73) |
| H21 | CGCACAGATGCGTAAGGAG | Reverse, to linearize pEWL69 (pEWL73) |
| EWL341 | CTCCTTACGCATCTGTGCG | Forward, to amplify 5’-UTR and RBS (pEWL73) |
| EWL345 | TAAAAATCCTTTGGGAAACGCTAGCGTAGGTTTCCGCACCGAG | Reverse, to amplify 5’-UTR and RBS (pEWL73) |
| EWL440 | GAGTCAAGACGCACACCTCA | Forward, to linearize pCRD206 (pEWL85) |
| H337 | ACCTTGAAATAGATAACCTCCTGCTACC | Reverse, to linearize pCRD206 (pEWL85) |
| EWL438 | GAGGTTATCTATTTCAAGGTAAGCGCCTCATCAGCGGTAA | Forward, to amplify *cre* (pEWL85) |
| EWL439 | TGAGGTGTGCGTCTTGACTCCGAAGCCGCACGTCATCTAG | Reverse, to amplify *cre* (pEWL85) |
| H887 | AATAAATCCTGGTGTCCCTGTTGG | Forward, to linearize pEWL85 vector (pEWL89) |
| H1003 | TCAGAATTGGTTAATTGGTTGTAACACTGGC | Reverse, to linearize pEWL85 vector (pEWL89) |
| EWL460 | GCCAGTGTTACAACCAATTAACCAATTCTGA | Forward, to amplify apramycin resistance cassette (pEWL89) |
| H19 | ATCCAACAGGGACACCAGG | Reverse, to amplify apramycin resistance cassette (pEWL89) |
| BH438 | ATATATGCGGCCGCATAT | Forward, for site-directed mutagenesis to delete BamHI site on pACM246 (pEMH119) |
| BH439 | ATATCATGCACCATTCCTTG | Reverse, for site-directed mutagenesis to delete BamHI site on pACM246 (pEMH119) |
| BH440 | GGATCCCTAGGTGCCTGGCGGCAG | Forward, for site-directed mutagenesis to delete *eGFP* and insert BamHI site at MCS on pEMH119 (pEMH120) |
| BH441 | ATGTAAAAAATCCTTTCGCTAGCAAATTGTTATCC | Reverse, for site-directed mutagenesis to delete *eGFP* and insert BamHI site at MCS on pEMH119 (pEMH120) |
| BH586 | GCGAAAGGATTTTTTACATGATGACTCGACGTCTACATG | Forward, to amplify *HA-rsdA* from pEMH169 for insertion into BamHI/AvrII-digested pEMH120 (pEMH193) |
| BH664 | GCGCTACTGCCGCCAGGCACCTAAGCATAATCGGGCAC | Reverse, to amplify *HA-rsdA* from pEMH169 for insertion into BamHI/AvrII-digested pEMH120 (pEMH193) |
| BH41 | AAGTCCGCCATGCCCGAA | Forward, to linearize pTGR5 backbone to delete *lacI*, P*_tac_*_,_ and *eGFP* (pEMH3) |
| BH42 | TGAGCGCAACGCAATTAATGTAAG | Reverse, to linearize pTGR5 backbone to delete *lacI*, P*_tac_*_,_ and *eGFP* (pEMH3) |
| BH43 | CATTAATTGCGTTGCGCTCAAAGCGCCTCATCAGCGGT | Forward, to amplify P*_sod_* and native RBS (pEMH3) |
| BH44 | CCTTCGGGCATGGCGGACTTGGGTAAAAAATCCTTTCGTAGGTTTCC | Reverse, to amplify P*_sod_* and native RBS (pEMH3) |
| BH148 | TGACCTAGGTGCCTGGCG | Forward, to delete native 6x His tag from pEMH6 (pEMH25) |
| BH149 | GGGTAAAAAATCCTTTCGTAGGTTTCC | Reverse, to delete native 6x His tag from pEMH6 (pEMH25) |
| BH71 | TGCGGTATTTCACACCGCATA | Forward, diagnostic primer for pTGR5-based vectors |
| BH72 | TCGCCCTCGAACTTCACCTC | Reverse, diagnostic primer for pTGR5-based vectors |
| BH250 | CCGGCGGATTTGTCCTACTCA | Reverse, diagnostic primer for pTGR5-based vectors |
| BH283 | TGGATGAACTGTACAAGTAATGACCTAGGTGCCTGGCG | Forward, to linearize pEMH25 for insertion of P*cgp_2320*::*mScarlet* gBlock (pEMH90) |
| BH315 | TGGTTTGAACGTTACACACCTGAGCGCAACGCAATTAATGTAAG | Reverse, to linearize pEMH25 for insertion of P*cgp_2320*::*mScarlet* gBlock (pEMH90) |
| BH443 | TGGTCTGGTGTCAAAAATAATGACCTAGGTGCCTGGCG | Forward, to linearize pEMH90 to delete *mScarlet* (pEMH121) |
| BH444 | TGGTCTGGTGTCAAAAATAATGACCTAGGTGCCTGGCG | Reverse, to linearize pEMH90 to delete *mScarlet* (pEMH121) |
| BH445 | ATGAGATCTACCATGATTACG | Forward, to amplify *lacZ* (pEMH121) |
| BH446 | TTATTTTTGACACCAGACC | Reverse, to amplify *lacZ* (pEMH121) |
| BH752 | GTTCTTCTGAGCGGGACTCTGGGGTTCG | Forward, to amplify pEMH121 vector (pEMH304) |
| BH753 | GTTCAATCATGCGAAACGATCCTCATCCTG | Reverse, to amplify pEMH121 vector (pEMH304) |
| BH754 | ATCGTTTCGCATGATTGAACAAGATGGATTG | Forward, to amplify kanamycin-resistance cassette (pEMH304) |
| BH755 | AGAGTCCCGCTCAGAAGAACTCGTCAAG | Reverse, to amplify kanamycin-resistance cassette (pEMH304) |
| BH124 | AAGTCCGCCATGCCCGAA | Forward, to amplify pEMH6 vector (pEMH117) |
| BH125 | GGGTAAAAAATCCTTTCGTAGGTTTCC | Reverse, to amplify pEMH6 vector (pEMH117) |
| BH126 | TACGAAAGGATTTTTTACCCATGGATCTTTCCCTTCTC | Forward, to amplify *porH* (pEMH117) |
| BH127 | CCTTCGGGCATGGCGGACTTTTAGGAAGAGAAGTTATCCAG | Reverse, to amplify *porH* (pEMH117) |
| BH151 | CACCACCACCACCACCAC | Forward, to use site-directed mutagenesis of pEMH117 to delete downstream region prior to native 6x His tag in order to fuse native 6x His tag onto *porH* (pEMH27) |
| BH152 | GGAAGAGAAGTTATCCAGATTCTCG | Reverse, to use site-directed mutagenesis of pEMH117 to delete downstream region prior to native 6x His tag in order to fuse native 6x His tag onto *porH* (pEMH27) |
| BH148 | TGACCTAGGTGCCTGGCG | Forward, to use site-directed mutagenesis to delete native 6x His tag from pEMH117 to match pEMH25 vector (pEMH26) |
| BH150 | GGGTAAAAAATCCTTTCGTAGGTTTCC | Reverse, to use site-directed mutagenesis to delete native 6x His tag from pEMH117 to match pEMH25 vector (pEMH26) |
| BH836 | ACAGGAAACAGAATTAATTAACGAAAGGATTTTTTACCCATGG | Forward, to amplify *porH-His* form pEMH27 for insertion into HindIII/EcoRI-digested pFSC (pEMH306) |
| BH837 | CCGCCAAAACAGCCAAGCTGTCAGTGGTGGTGGTGGTG | Reverse, to amplify *porH-His* form pEMH27 for insertion into HindIII/EcoRI-digested pFSC (pEMH306) |
| BH967 | TTTAAGTTTAAGTTGTAATCGAGCTGAAAGGCTGAGGCCTCAATATGGAATCCGTGATGGTAACTTCACG | Forward, recombineering primer for *pks* to amplify from pEWL74 |
| BH968 | AGTGGTGTTACTCAACTTCCGTCAGCTCTCCGTTACTAATTCTTCCGAGAAGCCGTCAATTGTCTGATTC | Reverse, recombineering primer for *pks* to amplify from pEWL74 |
| ACM206 | TCGTAGCCCTGGTTAGTGTC | (1) |
| ACM207 | ACGGTCTTGCAGAAGCATCC | (1) |
| BH970 | TCCAGGGGTTGCTGAAAAATCCTCGCATAGATTTAATCTAGATCCTCAAGAGCCGTCAATTGTCTGATTC | Forward, recombineering primer for *cmpL1* to amplify from pEWL74 |
| BH971 | TGGGCAGCATGTGTGGCCATATCCAGTGATGGAGGTGGACAATGCTGGATTCCGTGATGGTAACTTCACG | Reverse, recombineering primer for *cmpL1* to amplify from pEWL74 |
| BH1057 | AACAAGCGCCGCATCATCCAGT | Forward, diagnostic primer for *cmpL1* |
| BH1058 | GATGTAGGCGGCTTCGATGG | Reverse, diagnostic primer for *cmpL1* |
| BH1080 | CGTGGGCGATTCGTCGTTCAGAGGTCCAGGGTTAAAGCTTCGTGAAATGGTCCGTGATGGTAACTTCACG | Forward, recombineering primer for *cmpL2* to amplify from pEWL74 |
| BH1081 | CGCGCTAAAAGGAGCACTCATTAAACTTCGGCCCTTTCTGGGCTATTTATAGCCGTCAATTGTCTGATTC | Reverse, recombineering primer for *cmpL2* to amplify from pEWL74 |
| BH1082 | TGCGCACGCCTTGACCACG | Forward, diagnostic primer for *cmpL2* |
| BH1083 | TCAAGTTCACGCCGTCAACGG | Reverse, diagnostic primer for *cmpL2* |
| BH1076 | AAGCCCAGAAGAACAGTCAACTCCTAGATTAAAGGATAATCGTGGCGAAATCCGTGATGGTAACTTCACG | Forward, recombineering primer for *cmpL3* to amplify from pEWL74 |
| BH1077 | CCCTCACCTTCAACATCAACGTTGGGAAGAATCTTATCCAACCACTTAGGAGCCGTCAATTGTCTGATTC | Reverse, recombineering primer for *cmpL3* to amplify from pEWL74 |
| BH1078 | ATTGGCCCATATTTACCCTTGC | Forward, diagnostic primer for *cmpL3* |
| BH1079 | CCAACACCTGCACCCATAACC | Reverse, diagnostic primer for *cmpL3* |
| BH971 | TGGGCAGCATGTGTGGCCATATCCAGTGATGGAGGTGGACAATGCTGGATTCCGTGATGGTAACTTCACG | Forward, recombineering primer for *pptA* to amplify from pEWL74 |
| BH972 | TTCAGCTCACCCAGGTATCCTCTCCATCCAGTTCAAGTCACTGCAGTCGCAGCCGTCAATTGTCTGATTC | Reverse, recombineering primer for *pptA* to amplify from pEWL74 |
| BH959 | CTGGATTTCCTATGCATTAGATGCAGG | Forward, diagnostic primer for *pptA* |
| BH960 | CACCAGCACCGATGATGCAGAAGG | Reverse, diagnostic primer for *pptA* |
| BH973 | TTAACCACGAACGTTTTAAAGAAGCCACGAAGGAGCCTGACATGGCGTTATCCGTGATGGTAACTTCACG | Forward, recombineering primer for *cmrA* to amplify from pEWL74 |
| BH974 | GTGTTGTTTTCAGATTCAGACACTTTTAAAAACTAACCCATCTTCTTATAAGCCGTCAATTGTCTGATTC | Reverse, recombineering primer for *cmrA* to amplify from pEWL74 |
| BH1055 | TAAACCCATCGCAGGGGCG | Forward, diagnostic primer for *cmrA* |
| BH1056 | ACGCAATCGCGCAATCGCC | Reverse, diagnostic primer for *cmrA* |
| BH1070 | AGCTGATGAGCACTGTGAATAAACTCAGGAGTTAAATCCTTATGACCCCGTCCGTGATGGTAACTTCACG | Forward, recombineering primer for *mmpA* to amplify from pEWL74 |
| BH1071 | AGTGGGACACCGCGGGAGCGCAGTTCGAGGAACTACTTCTTGGTCTTGCGAGCCGTCAATTGTCTGATTC | Reverse, recombineering primer for *mmpA* to amplify from pEWL74 |
| BH1074 | GCTGGCGGGGTCGACG | Forward, diagnostic primer for *mmpA* |
| BH1075 | GGTGGGTGAGTTTTTGGCGGAT | Reverse, diagnostic primer for *mmpA* |
| BH999 | ACCTCAAACGCCTAGCCCGCATCATGCCCGCATACTGGGCAACGGTCATTTCCGTGATGGTAACTTCACG | Forward, recombineering primer for *tmaT* to amplify from pEWL74 |
| BH1000 | AAATTTCCACTAAATAAAGGCAACCCGAGCAGTGGGAACACAATCGTCAGAGCCGTCAATTGTCTGATTC | Reverse, recombineering primer for *tmaT* to amplify from pEWL74 |
| BH941 | CGACAACGCAGGATGGTCC | Forward, diagnostic primer for *tmaT* |
| BH942 | TCGCATCGACTGTGGCCCC | Reverse, diagnostic primer for *tmaT* |
| BH977 | AGCGTTTCCGCCCCATCGCTGCAGGAGTCCTGCCAGTAGGAATGGCATACTCCGTGATGGTAACTTCACG | Forward, recombineering primer for *ubiA* to amplify from pEWL74 |
| BH978 | GCGAGGAAAGACAGTCCGATGGATAGTGCAATGAGCGCCACGGCCATGCCAGCCGTCAATTGTCTGATTC | Reverse, recombineering primer for *ubiA* to amplify from pEWL74 |
| BH1090 | TCTTCACATGCGACCTCGACAAC | Forward, diagnostic primer for *ubiA* |
| BH1091 | ACCGTTAGATGCGGCGCGAG | Reverse, diagnostic primer for *ubiA* |
| BH965 | GCAATGCTCGTTGAGTATGAAAGACTTCTATCATGATTAACACCTCTGAATCCGTGATGGTAACTTCACG | Forward, recombineering primer for *aftA* to amplify from pEWL74 |
| BH966 | CGTTTGGTGAGGAATTACTCATTGTGCGTTACCACCACGAAAGGTCCCACAGCCGTCAATTGTCTGATTC | Reverse, recombineering primer for *aftA* to amplify from pEWL74 |
| BH1116 | GGATCCGGTGCCAACGTATTG | Forward, diagnostic primer for *aftA* |
| BH117 | TCAACTACATCTGACACGTTGATC | Reverse, diagnostic primer for *aftA* |
| BH1005 | TAATGGGGGCCATCAACTAGACTCGATCAACGTGTCAGATGTAGTTGAGTTCCGTGATGGTAACTTCACG | Forward, recombineering primer for *emb* to amplify from pEWL74 |
| BH1006 | CAGTACTGAAGGTCTTATTCATCTACCTTCATATGCCCTGGATTCCAGAGAGCCGTCAATTGTCTGATTC | Reverse, recombineering primer for *emb* to amplify from pEWL74 |
| BH1112 | TCCAAACGTGCGCTTC | Forward, diagnostic primer for *emb* |
| BH1113 | TTGGTGTCGTGCTCGC | Reverse, diagnostic primer for *emb* |
| BH1124 | GGCCACATGCGGTGCTAGCATGTGGCCTCATGACGTTTAGCCCCCAGCGTTCCGTGATGGTAACTTCACG | Forward, recombineering primer for *aftB* to amplify from pEWL74 |
| BH1125 | GGGTAGTTATCACAGCCCAATGATTTGCGAATAAGTGTTTACTGAGAGCTAGCCGTCAATTGTCTGATTC | Reverse, recombineering primer for *aftB* to amplify from pEWL74 |
| ACM410 | CGTTGACGTTGAATCCACCG | Forward, diagnostic primer for *aftB* |
| ACM411 | GTAGTGTTCTGCTTCGGTGC | Reverse, diagnostic primer for *aftB* |
| BH1001 | GCATCGGCAACGCGGTTGCATGGCCGTTGGCCATGTTGTTGATGGCGCATTCCGTGATGGTAACTTCACG | Forward, recombineering primer for *aftC* to amplify from pEWL74 |
| BH1002 | TTTGAAGTCTGTCATGCTGTCCTCTCAAGATGGTCGTGCGTTGGATCAGTAGCCGTCAATTGTCTGATTC | Reverse, recombineering primer for *aftC* to amplify from pEWL74 |
| BH947 | TGGGCCCATTGCATGTTTATGGATCTA | Forward, diagnostic primer for *aftC* |
| BH948 | TGCAGATCTGATTAAGGTTCTTCCTTA | Reverse, diagnostic primer for *aftC* |
| BH975 | TTCACTCGGCGCATTTCTATGTCTGGATTGTGCTGGGTTTTGTGGTGTTTTCCGTGATGGTAACTTCACG | Forward, recombineering primer for *aftD* to amplify from pEWL74 |
| BH976 | GCCTAGAATCGTCAGAATGAACTGACACCCATTTAGCGCTTTGGAGGCCTAGCCGTCAATTGTCTGATTC | Reverse, recombineering primer for *aftD* to amplify from pEWL74 |
| BH953 | CGGTGCGGGAGGGGTG | Forward, diagnostic primer for *aftD* |
| BH954 | GTGCGTGGAAGTGGCGTTTT | Reverse, diagnostic primer for *aftD* |
| ACM548 | GCTTGCGGATTCTGTCAACC | Forward, recombineering primer for *mptA* upstream region to amplify from pACM246 |
| ACM549 | GAAAAATAAACAAATAGGGGTTCCGCGACGCGTCTACTACATCCGAC | Reverse, recombineering primer for *mptA* upstream region to amplify from pACM246 |
| ACM550 | CATTTGATGCTCGATGAGTTTTTCTAATTGCATAAGGTAAACCGCCAC | Forward, recombineering primer for *mptA* downstream region to amplify from pACM246 |
| ACM551 | GGTCAAGAAGGTGCTTTCATTCC | Reverse, recombineering primer for *mptA* downstream region to amplify from pACM246 |
| ACM552 | ATGCGTACTGAGGTTATTGG | Forward, diagnostic primer for *mptA* |
| ACM553 | GATACCTGTCCTCTAAAACG | Reverse, diagnostic primer for *mptA* |
| ACM554 | GTCTAGGTGCCTGCGCACTC | Forward, recombineering primer for *mptB* upstream region to amplify from pACM246 |
| ACM555 | GAAAAATAAACAAATAGGGGTTCCGCGAGTGTCGAGAAATTGGCGTAG | Reverse, recombineering primer for *mptB* upstream region to amplify from pACM246 |
| ACM556 | CATTTGATGCTCGATGAGTTTTTCTAATTACACTGATCAACTGTGACTACTG | Forward, recombineering primer for *mptB* downstream region to amplify from pACM246 |
| ACM557 | GAGGAAGATAATCTCAGGGCGACC | Reverse, recombineering primer for *mptB* downstream region to amplify from pACM246 |
| ACM558 | TTTATCAGTAAGCCGATAAG | Forward, diagnostic primer for *mptB* |
| ACM559 | AGCTCCCACACCATGTTGCG | Reverse, diagnostic primer for *mptB* |
| ACM560 | GAACTTGGCTAAATTATGACGCG | Forward, recombineering primer for *mptC* upstream region to amplify from pACM246 |
| ACM561 | GAAAAATAAACAAATAGGGGTTCCGCGGAAAAATAGTGTATCCGTATCCAG | Reverse, recombineering primer for *mptC* upstream region to amplify from pACM246 |
| ACM562 | CATTTGATGCTCGATGAGTTTTTCTAAGCCTTACAGTCCGACAGCCTC | Forward, recombineering primer for *mptC* downstream region to amplify from pACM246 |
| ACM563 | CCAGGCACGGTGTTGTCTTC | Reverse, recombineering primer for *mptC* downstream region to amplify from pACM246 |
| ACM564 | AAGTTTTCTCCCAACCTATG | Forward, diagnostic primer for *mptC* |
| ACM565 | ATGAGGTTGATGTTGTTGTC | Reverse, diagnostic primer for *mptC* |

**BIBLIOGRAPHY**

1. McKitterick AC, Bernhardt TG. 2022. Phage resistance profiling identifies new genes required for biogenesis and modification of the corynebacterial cell envelope. *Elife* 11:e79981.
