# Supplementary material for: The conserved σD envelope stress response monitors multiple aspects of envelope integrity in corynebacteria
Source: PLoS Genet. 2024 Jun 3;20(6):e1011127. doi: 10.1371/journal.pgen.1011127 (PMC11175481; doi:10.1371/journal.pgen.1011127)
Supplement: Attachment — Submitted filename: ResponsetoReviewers-final.docx [file pgen.1011127.s017.docx]

**Response to Reviewers**

**Reviewer #1:**

**(1) Are beta gal assays complementable? This is a relatively minor concern as there are lots of different mutants and treatments to test each hypothesis.**

**RESPONSE:** We agree with the reviewer that complementing the genetic deletions would be ideal. However, given the number of genetic loci involved, such a complementation strategy is not feasible. As the reviewer mentions, complementation is not critical in this case given that similar phenotypes obtained with many different deletion alleles such that they serve as controls for each other. We attempted to complement the *aftA::kan* and *emb::kan* phenotypes due to their arrangement in what appears to be an operon. However, because the mutants grow so poorly, it was not possible to manipulate the strains for complementation despite several attempts. We have therefore included language in lines 556-558 to indicate that it is possible that the *aftA::kan* allele is polar on the downstream *emb* gene.

**(2) First paragraph of Discussion is helpful, especially the last sentence, and I wonder if the authors could have a similar sentence somewhere at the end of the Introduction to help orient the reader and set expectations.**

**RESPONSE:** As suggested, we have added text to the Introduction (lines 118-119) to help set reader expectations.

**(3) Many of the deletions are in cell envelope genes that seem like they should be essential, at least by analogy to other organisms. Can the authors address this point somewhere?**

**RESPONSE:** The reviewer is correct that many of the genes we deleted are essential in other members of the Corynebacteriales/Mycobacteriales such as *Mtb*. Herein lies the power of Cglu as a model system as these genes are not strictly essential in this organism. We have highlighted this important point in the Introduction in lines 89-91.

**(4) Fig. 4 legend missing panels (E) and (F).**

**RESPONSE:**The description for panels 4E-F are included in the figure legend, grouped with the description for Figures 4B-C (Figure 4 legend, line 380).

**Reviewer #2:**

**(1) Overall, while the data are very convincing on their own, most figures would benefit from ANOVA statistical analysis, as in Figure 1A. For example, there appears to be some intermediate activation of the lacZ reporter in Figure 4F for loss of aftB.**

**RESPONSE:** We have added ANOVA statistical analysis for the β-galactosidase assays shown in Figure 4B-C, E-F and Figure 5C-D. Descriptions of these analyses have been added to the figure legends, where appropriate (Figures 4-5 legends, lines 381-383, lines 462-463).

**(2) The logic on Page 8 is confusing around the unexpected decrease in signal upon expression of the second copy of cmt1. I suggest rewriting this section to clarify the logic of the experiments and the conclusion. In addition to this, the statements around the PorH proteoforms in Figure S3 are not convincing. Claiming a difference in the upper two proteoforms for ΔsigD in S3B is difficult without loading controls, replicates, and quantification. I suggest either softening this claim or providing more evidence. Overall, the conclusions from Figures 2D and S3 feel a little disjointed. The rest of the paper was clearly written and the experiments were explained nicely.**

**RESPONSE:** We have rewritten this section (lines 225-245) in order to clarify the motivation behind the experiments and the results. This includes a statement regarding the unexpected reduction in PorH-His surface exposure upon Cmt1 overexpression as well including additional immunoblot replicates to bolster our statement regarding the PorH proteoforms (revised Figure S3, legend of Figure S3 line 1125). We hope that our changes have addressed the confusion regarding this section of the paper.

**(3) The figure call to Figure S4 mentions inductions at different stages of growth, but this is not shown.**

**RESPONSE:**We apologize for this error. This phrase has been removed from the sentence on line 314.

**Page 18: missing a period at sentence ending “…defects in AG biosynthesis that promote sD activation”**

**RESPONSE:** We have fixed this grammatical issue.

**Reviewer #3:**

**Major comments:**

**(1) The authors suggest that sigmaD responds to “loss of the arabinan chain” and, in the Discussion, they further speculate that “RsdA might use its long, disordered periplasmic domain of approximately 160 amino acids to detect AG defects”. However, it seems equally likely that RsdA senses the accumulation of lipid-linked galactan core in the inner membrane. The authors should provide additional experimental evidence to support this speculation or tone down with their claim that RsdA is sensing the loss of arabinan chain.**

**RESPONSE:** We agree with the reviewer that RsdA could be sensing an accumulation of galactan core in the inner membrane. We do not favor this possibility as there are no data to suggest that the galactan core of AG accumulates in the inner membrane in the absence of the arabinan chain. However, we have included this as an alternative model in the Discussion (lines 577-579).

**(2) Without providing the complemented strain for the *emb::kan* and *aftA::kan* strains, it remains possible that sigmaD activation is due to a polar effect of *emb* or *aftA* gene deletion. Since RsdA may be sensing inner membrane defect (such as the absence of TMM as the authors suggest), it is even possible that RsdA is sensing the absence of Emb or AftA proteins rather than the defects in**

**arabinans. These mutants should be complemented minimally by wildtype genes, and ideally also by catalytically inactive versions of the corresponding genes.**

**RESPONSE:** We appreciate this comment and agree that it would be ideal to complement the *aftA::kan* and *emb::kan* mutations. We made numerous attempts to complement the *aftA::kan* and *emb::kan* mutations but were unsuccessful due to the extremely poor growth of the strains preventing the isolation of transformants with an *aftA* containing plasmid for complementation. Given this difficulty, we have revised the text describing these mutants to mention the possibility that the *aftA::kan* mutation may be polar on the downstream *emb* gene (lines 556-558). Because *emb* is downstream of *aftA*, the *emb::kan* allele is unlikely to be polar on *aftA*.

**3. Similarly, ethambutol may have secondary side effects on the inner membrane homeostasis. If *emb::kan* is complemented by ethambutol-resistant version of emb, and if ethambutol does not induce sigmaD activation in the complemented strain, that would strengthen the authors’ claims. Minimally, the authors should discuss these alternative possibilities.**

**RESPONSE:** We agree that the suggested experiment would be valuable addition to this study. As detailed above, technical limitations have prevented complementation of the *emb::kan* allele. We now mention potential EMB side effects on inner membrane homeostasis as an alternative model of σ^D^ activation in the Discussion (lines 556-558).

**(4) Provide the gene ID of ProtX. Provide how the mutant was generated, including the plasmids and oligos used to generate it.**

**RESPONSE:** We apologize for this accidental omission. We have added the gene ID for ProtX (Figure S3 legend, line 1121) and have included plasmids/oligos used to generate the strain in Tables S2-4.

**5. The authors stated “In the absence of Cmt1, PorH-His cannot be O-mycoloylated, leading to a dramatic decrease in PorH levels in the MM (6–8, 12). As anticipated, *cmt1::zeo* cells expressing PorH-His showed no surface fluorescence (Figure 1A)”. This statement is misleading as the authors later show that PorH is not even expressed at all in a cell lysate of the *cmt1* mutant (Fig. S3). If the authors truly intend to make the claim that PorH level is reduced in the MM, but accumulating elsewhere, there should be supporting evidence for the claim.**

**RESPONSE:** Previous work has shown that in the absence of Cmt1, PorH (and other porins) are secreted from the cell and can be found in the supernatant (Carel *et al, PNAS*, 2017). Thus, PorH is still expressed in *cmt1* null cells, but is not properly localized to the MM.

**Minor Comments:**

**(1) In Corynebacteria, TMM and TDM should be described as trehalose monocorynomycolate (TMCM) and TDCM.**

**RESPONSE:** We have changed TMM and TDM to TMCM and TDCM, respectively, throughout the manuscript and supplementary material.

**(2) In Fig. 1, the Cmt1 mutant should be complemented to confirm that the observed phenotype is indeed due to the deletion of cmt1.**

**RESPONSE:** We have complemented the *cmt1* mutant cells by expressing an ectopic copy and measured PorH-His surface exposure by flow cytometry (Figure S2).

**(3) On page 4, Line 19, the argument “... the biogenesis of which is targeted by front-line anti-mycobacterial therapies." needs references.**

**RESPONSE:** We have added two references for this statement (line 99).

**(4) On page 6, Line 13, Cmt1 was not annotated in the cited papers. Indicate the gene number of Cmt1 to help orient readers.**

**RESPONSE:** We have included the gene ID for *cmt1* on the line in which it is first mentioned (line 108).

**(5) On page 10, line 2, confirm *porH* overexpression through immunoblotting. Otherwise, the argument “No induction was observed when the MOMP was overproduced” cannot be supported.**

**RESPONSE:** For the PorH overexpression, the cells are ectopically expressing a second, tagged copy of PorH (PorH-His) in addition to the native copy. We have included immunoblot analysis of these strains demonstrating that PorH-His is expressed. This data is included as Figure S5 and referenced in the text on line 322. All other supplementary figure numbering has been adjusted accordingly.

**(6) In Figure 2B, modify the graph label to make it clear that *cgp_0697* is *rsdA*.**

**RESPONSE:** Figure 2B has been updated to edit this nomenclature.

**(7) In Figure 3D, indicating the expected molecular weight of the full-length and cleaved protein would be helpful for readers.**

**RESPONSE:**The cleavage sites on RsdA for MarP and Rip1 are currently unknown, so we do not know the expected molecular weight for the cleaved products. Figure 3D includes the annotated molecular weight ladder for the immunoblot shown.

**(8) Regarding Figure S9, it appears that AU1235 increased the relative fluorescence of 6TMR-Tre. Please clarify why this is happening.**

**RESPONSE:** It is unclear why we observe an increase in 6TMR-Tre signal in wild-type cells treated with AU1235. We think that this is an interesting result as well but believe discussion is outside of the scope of the paper.

**(9) Indicate the panel when referring to Fig. S3 in the text.**

**RESPONSE:** We have clarified our discussion of Figure S3 in the text (lines 225-245) including referencing the specific panels (lines 233 and 238).
